# Limited water stress modulates expression of circadian clock genes in *Brachypodium distachyon* roots

**DOI:** 10.1038/s41598-022-27287-4

**Published:** 2023-01-23

**Authors:** Magdolna Gombos, Nóra Hapek, László Kozma-Bognár, Gábor Grezal, Zoltán Zombori, Edina Kiss, János Györgyey

**Affiliations:** 1 Institute of Plant Biology, BRC-Biological Research Centre, Szeged, Hungary; 2 Institute of Biochemistry, BRC-Biological Research Centre, Szeged, Hungary; 3 Department of Genetics, Faculty of Science and Informatics, University of Szeged, Szeged, Hungary

**Keywords:** Plant sciences, Plant molecular biology, Plant stress responses

## Abstract

Organisms have evolved a circadian clock for the precise timing of their biological processes. Studies primarily on model dicots have shown the complexity of the inner timekeeper responsible for maintaining circadian oscillation in plants and have highlighted that circadian regulation is more than relevant to a wide range of biological processes, especially organ development and timing of flowering. Contribution of the circadian clock to overall plant fitness and yield has also long been known. Nevertheless, the organ- and species-specific functions of the circadian clock and its relation to stress adaptation have only recently been identified. Here we report transcriptional changes of core clock genes of the model monocot *Brachypodium distachyon* under three different light regimes (18:6 light:dark, 24:0 light and 0:24 dark) in response to mild drought stress in roots and green plant parts. Comparative monitoring of core clock gene expression in roots and green plant parts has shown that both phase and amplitude of expression in the roots of *Brachypodium* plants differ markedly from those in the green plant parts, even under well-watered conditions. Moreover, circadian clock genes responded to water depletion differently in root and shoot. These results suggest an organ-specific form and functions of the circadian clock in *Brachypodium* roots.

## Introduction

Intrinsic oscillations with a period close to the 24-h long rotation time of the Earth—called circadian rhythms—provide temporal organization and precise timing of biological processes in every living organism from cyanobacteria to mammals. Proper adjustment of these rhythms is provided by daily fluctuation of environmental cues, such as light/dark or warm/cold cycles, as input signals. The fundamental role of daily rhythms in biological processes is widely known, and the importance of the circadian clock in fitness optimization through temporal synchronization of gene expression, metabolism and physiology to predictable environmental changes has long been a central issue in plant biology since Androsthenes described the sleeping movement of leaves as the first recorded evidence for daily rhythms in plants more than two millennia ago^1^. The molecular network of the inner clockwork that establishes daily oscillations in plants has been thoroughly reported in *Arabidopsis thaliana*. In *Arabidopsis,* the heart of the main oscillator consists of three interlocking regulatory loops: a morning-phased, a central and an evening-phased loop^2^. In brief, the day starts with an expression peak of two partially redundant MYB-domain transcription factors, LATE ELONGATED HYPOCOTYL (LHY) and CIRCADIAN CLOCK ASSOCIATED1 (CCA1). In the afternoon and at night, LHY and CCA1 are down-regulated by an evening-phased transcriptional repressor PSEUDO-RESPONSE REGULATOR1/TOC1 (PRR1/TOC1—TIMING OF CAB). Interplay between LHY, CCA1 and TOC1 forms the first loop in the center of the circadian clock. The other key loop consists of TOC1-related PRR proteins (PRR3, 5, 7 and 9) expressed sequentially after dawn in the following order: PRR9, PRR7, PRR5 and PRR3^3^. The overlapping expression pattern of these PRR repressor proteins establishes sequential and extended regulation of TOC1 expression, stability and nuclear transport throughout the day^4^. The 3rd cycle is formed by the evening-phased components EARLY FLOWERING 3 (ELF3), EARLY FLOWERING 4 (ELF4) and LUX ARRHYTHMO (LUX), which interact to form a transcriptional repressor complex called evening complex (EC)^5^. LHY and CCA1 are released from the inhibition late at night by the members of the evening complex, thus allowing elevated expression of LHY and CCA1 the following morning. LHY and CCA1 subsequently inhibit TOC1 expression. At the same time, F-box proteins, such as GIGANTHEA (GI) in complex with LKP2 (LOV KELCH PROTEIN2) promote light-dependent degradation of TOC1 and increase the amplitude of the oscillation so that the circadian clock can make a new turn^2,6^. This simplistic sketch of the circadian oscillator only represents a framework, which has been widened with many circadian-associated components in the last few decades. To date, more than 20 clock-associated genes have been identified and many more are yet to be incorporated in the model, resulting in a more complex and robust network complemented with “repressilator” circuits^6^.

Based on transcriptome sequencing, approximately 30% of total transcripts are clock-regulated under favorable environmental conditions in *Arabidopsis,* thus supporting the general view that the circadian clock might be the master conductor of plant gene expression^7^. Many genes central to essential biological processes, such as flowering time, photosynthesis, biosynthesis and signaling of phytohormones, growth control and metabolic activities, can be found on the output side of the circadian clock. Unsurprisingly, a wide range of studies on the plant stress transcriptome have demonstrated that even responses to different environmental stresses are also shaped by the time of day^8,9^. The interplay between biotic stresses and diel cues is well-established. In the case of biotic stresses, circadian gating of responses enables defense gene expression during the day, but it dampens at night, thus enabling resources to be saved for growth^10,11^. With the knowledge of the interplay between plant stress hormones (e.g. jasmonate and abscisic acid) and the circadian clock, rhythmic regulation of many aspects of abiotic stress responses might not be surprising either^12,13^. Datasets from osmotic stress, salinity, temperature changes and water deprivation have indicated that a high number of abiotic-stress genes fall under circadian control^14–16^.

Studies reporting time-shaped regulation of *Arabidopsis* transcriptome and growth under mild drought pointed to the gating function of the circadian clock under drought conditions similar to circadian control of biotic stress responses. With an unfavorable water supply, growth is shut down during the day and preserved during the night, when activation of drought responses is crucial^17,18^. When drought sets in, plants react with a flexible way to reprogram growth to increase their chances of survival, but decrease yield. In accordance with physiological responses, such as stomata closure, growth arrest of young leaves etc., the time of day also strongly affects the extent, specificity and, in some cases, the direction of drought-induced changes in gene expression to save water and energy^17^. It is still unclear if changes in the diurnal expression pattern of drought responses result from altered circadian clock regulation or not, but the effect of the time of day on the drought response is beyond doubt. Nevertheless, many of the outputs have a feedback connection to the circadian clock and can serve as input signals, thus establishing a dynamic regulatory network based on continuous crosstalk between environmental sensing pathways and the circadian clock^19^.

Clock components are present in each plant cell, and their core function in precise timing and temporal separation of metabolic and developmental pathways are generally valid for each plant tissue. However, the outcome is merely organ-specific. Recent experiments on the plant circadian clock shed light on tissue-specific aspects and its hierarchical coupling in green parts of *Arabidopsis* and tobacco^20–22^. For example, CCA1 oscillation has been shown to have a longer period and lower amplitude in stomata guard cells and a different period in different leaf segments and in different whorls of the rosette^21^. The TOC1 oscillation phase was also different in the vasculature^23^. Observations on tissue-specific plant circadian clocks indicate that an asymmetric coupling of circadian clocks from vasculature to mesophyll cells and shoots might exist^24^. However, little attention has been devoted to roots so far. Although there are some indications of a limited number of clock components in the roots^24–27^, we know almost nothing about the importance of the circadian oscillator in that organ.

A detailed picture of the circadian clock has taken shape thanks to intensive research on the model dicot *Arabidopsis thaliana*. Studies on the circadian clock in other species and their evolutionary lineages suggest conservation of the circadian clock^28,29^. As limited water supply has the most severe impact on agriculture, the contribution of the circadian regulation to drought adaptation in cereals has aroused great public interest recently. Based on Simon and colleagues’ report, relative change in biomass production caused by altered water use efficiency (WUE) in different clock mutant *Arabidopsis* lines ranges from − 70% to + 80%. The impact of the circadian clock on long-term WUE and its significant relation to biomass production in *Arabidopsis* lead to the assumption that optimization of the circadian clock might be suited to improve crop productivity under drought conditions^30,31^.

Although circadian clock genes are largely conserved among grasses and eudicots, comparative studies suggest that direct translation of knowledge on the *Arabidopsis* circadian clock to other plant species, such as cereals, might not be straight-forward due to differences between monocots and *Arabidopsis* in clock gene evolution and in clock-controlled processes, such as growth rhythm and photosynthetic metabolism^28,32^. For example, in *Brachypodium distachyon*, the model species for temperate grasses and cereals^33^, phylogenetic studies have revealed different routes in the evolution of *LHY/CCA1*, the *PRR*-family and *EARLY-FLOWERING-LIKE* genes^28,34^. *Brachypodium* appears to have a single *LHY/CCA1* counterpart with two alternative transcripts (*LHY1.1* and *LHY1.2*). They differ in length due to a retained intron at the 5′ end of the *BdLHY1.2* transcript, resulting in a truncated protein isoform in the case of BdLHY1.2. As regards the structure, *BdLHY1.2* resembles the alternative splice variant of *AtCCA1* (*AtCCA1ß*)^35^. This AtCCA1β isoform has a protein domain required for dimerization but lacks the MYB DNA binding motif. It is assumed that AtCCA1β inhibits functional AtCCA1α activity competitively by forming nonfunctional CCA1α-CCA1β and CCA1β-LHY heterodimers^36,37^. A conserved intron retention event is assumed in the background that resulted in two transcript variants of *CCA1/LHY* mRNA in evolutionarily distant species, such as *Arabidopsis, Brachypodium*, maize (*Zea mays*), rice and poplar (*Populus trichocarpa*)^38^. Moreover, this intron retention is supposed to be associated with abiotic stress conditions, such as high light and cold in *Arabidopsis*^36,37^. Nevertheless, the presence of two different protein isoforms of LHY/CCA1 with different functions is yet to be confirmed in *Brachypodium*.

As regards the *PRR* family, the same number of clock-associated *PRR*s was identified in *Brachypodium* as in *Arabidopsis.* Although a single homologue of *TOC1/PRR1* is present in *Brachypodium*, it is difficult to distinguish between real counterparts to *AtPRR3, 5, 7* and *9*. There are two *Brachypodium* genes with equal similarity to *AtPRR3* and *AtPRR7,* and the same situation can be seen in the case of putative *Brachypodium* homologues of *AtPRR5* and *AtPRR9*. Thus *Brachypodium PRRs* are designated in the literature as *PRR37, PRR73, PRR59* and *PRR95*^28,34^. Considering the ambiguous sequence similarity, it is very likely that these *PRRs* of *Brachypodium* evolved on different routes compared to *Arabidopsis PRRs* and that they might not be one-to-one equivalents^28,34^.

In terms of the evening complex, a single orthologue of *LUX* and *GI* are known in *Brachypodium* and one *ELF3* homologue has been identified^28,34^*.* More intriguingly, *Brachypodium* seems to have no orthologue of *ELF4* in a strict sense. *Arabidopsis* has one *ELF4* gene and four *ELF4-like* genes (*EFLs*) that share the same conserved domain with an unknown function (DUF1313). Based on full-length amino acid sequence similarity ELF4 and ELF4-like proteins (EFLs) of *A. thaliana* are classified into two clades: an ELF4-related group, which includes AtELF4—the well-known circadian system-devoted member of the ELF4 family with an essential role in flowering time regulation—and AtEFL1 (EARLY FLOWERING4-like 1) and the other subclade of ELF4-like2/3/4 (AtEFL2/3/4)^39^. Although diverse functions of AtELF4 are known in great detail, the role of EFLs and their link to the circadian clock are still unclear^39,40^. In silico analyses have shown that ELF4 subgroup members are only found in dicot species, while ELF4-like2/3/4 subfamily members are widely found in higher plants^41^. ELF4-like sequences of grasses appear to be related to the ELF4/EFL1 lineage of *Arabidopsis*, but the proposed independent gene diversification in grasses makes it difficult to identify proteins with an ELF4-like function in monocots^39^. The evolutionary history of the *ELF4* family explains the lack of the *AtELF4* homologue form *Brachypodium* and the presence of three *ELF4-like* related genes (*ELF4-like3, ELF4-likeA* and *ELF4-likeB*)^28,34^. With regard to the lack of one-to-one orthologues of *Arabidopsis ELF4* and *CCA1* in monocot species which have been investigated so far, it is generally held that these genes are dicot or at least *Arabidopsis*-specific genes^28^.

In addition, estimates for the proportion of protein-coding genes that are controlled by the circadian clock generally range between 10 and 13% in many monocots that are significantly smaller compared to *Arabidopsis* (10.8% for maize^42^, 12.6% for rice^32^ and 11.5% for *Setaria viridis*^43^). For *Brachypodium,* MacKinnon et al. predicted that approximately 3% of the transcriptome is under circadian clock regulation^44^, with the notion that the outcome of these estimations may vary based on the analysis method applied and that the composition of rhythmic gene sets may differ depending on the entrainment conditions, light intensity, temperature range during the day etc. Nevertheless, overall reduction in cycling genes between monocots and *Arabidopsis* highlights evolutionary divergence in clock functions.

In this research we attempt to assess the response of core clock genes to modest water deprivation with a focus on roots in comparison to green plant parts by systematically monitoring the gene expression changes of clock genes with special reference to *Brachypodium distachyon*. As this is a descriptive study, we had no preconceptions of the expressional behavior of *Brachypodium* clock genes, especially in terms of drought stress. However, based on the previously reported conserved manner of clock gene expression in *Brachypodium* leaves under favorable conditions^44,45^, we also expect expression patterns of *Brachypodium* clock genes similar to those of their *Arabidopsis* counterparts in green plant parts under well-watered conditions. On the other hand, patterns of clock gene expression in roots were supposed to be different from that of green plant parts.

## Materials and methods

### Plant growth conditions and sample collection

For the experiments, we used the standard or reference diploid Bd21 *Brachypodium distachyon* inbred line. Seeds of Bd21 accession were obtained from the publicly available *B. distachyon* collection at the National Plant Germplasm System of the USDA-ARS, Pullman (source accession: PI 254867) (https://www.ars.usda.gov/npgs/)^46^ and were propagated for the experiment in a controlled greenhouse environment. Experimental plants were grown in an illumination- and temperature-controlled plant growth chamber in compliance with all the relevant safety regulations of our government research institute.

Prior to germination, the seeds were sown on wet soil for 5 days at 4 °C in darkness (stratification). After stratification, the seeds were planted in pots filled with a mixture of sand and perlite (2:1) (five seedlings in each pot). The plants were grown in the growth chamber with the following standard set-ups: 140 µmol m^−2^ s^−1^ light intensity of warm-white fluorescent lighting, 22 °C/19 °C light/dark temperature, 60–65% relative humidity. Water and nutrient supply was provided by irrigation with a 0.5% Hoagland solution. At the beginning of the experiments, the plants were grown with optimal water content [80% field capacity (FC) where a relative 100% FC of the sand:perlite mixture is 260–265 g/kg] for two weeks. To maintain the water and nutrient supply, the plants were watered from the top daily during this two-week period. After that, half of the population was grown without further watering until the pots reached 40% FC (5–6 days on average), and then moderate water depletion (40% FC) was maintained for a week prior to sampling by irrigation every second day up to 40% field capacity. The other half of the population was irrigated every second day up to 80% FC. During this two-week period, irrigation was carried out from the bottom of the pots using water-permeable drains placed at the center of the pots. Samples were collected 4 weeks after germination from plants grown under two different water conditions (80% FC and 40% FC) and three different light regimes. In the sampling period, the plants were at a mature vegetative developmental stage—at the stage of main stem elongation, when the tillering phase is completed and the flag leaves are visible but still rolled—immediately before booting (at the BBCH37-38 phenological growth stage according to the numerical system developed by Biologische Bundesanstalt, Bundessortenamt and CHemische Industrie)^47^.

Before sampling, the plants were grown under the standard long-day lighting for *Brachypodium* growth: 18 h light/6 h dark photoperiod (light from 6:00 to 24:00). Three different light conditions were used for the sample collection days: 18:6 light:dark (LD), 24:0 light (LL) and 0:24 darkness (DD). Temperature was constant under LL and DD light conditions (22 °C and 19 °C, respectively).

It is worth noting that the relative water content of the pots was monitored daily throughout the experiment by measuring pot weight. At the beginning of sampling, relative water content was around 75% FC in the well-watered (WW) pots and 38% FC in the stressed ones. At the final point of sampling, relative water content was reduced to 50% FC in WW and 30% FC in the stressed pots under LD. Loss of water in DD was only 10% in the WW pots and 5% in the stressed ones by the end of the sampling. However, the slope of the water-loss curve dipped steeply in constant light (LL). At the end of three-and-a-half days of continuous lighting (LL), the relative water content of the WW pots was around 20% FC and 8–9% FC in the stressed ones, thus indicating extensive evaporation and a gradual build-up of moderate drought stress at the end of the experiments.

The samples were harvested every four hours at the following time points: ZT2, ZT6, ZT10, ZT14, ZT18, ZT22, ZT26, ZT30, ZT34, ZT38, ZT42, ZT46, ZT50, ZT54, ZT58, ZT62, ZT66, ZT70, ZT74 and ZT78. ZT (zeitgerber time or circadian hours) is defined as hours spent after providing an external light signal to entrain the circadian clock—in our experimental set-up ZT0 is light on (6:00).

In each light regimes, water conditions and time points the whole root system (including the primary root, lateral roots and shoot-borne crown roots) and the total aerial parts (green plant parts including the stems, stem nodes and leaves—shoots or green plant parts hereafter) of five plants were harvested in two biological replications. Low intensity green light (wavelength: 525 nm) was used to harvest in the dark. Watering was suspended during the three-and-a-half days of sample collection. The samples were freshly frozen immediately in liquid nitrogen and then stored at − 80 °C.

### RNA isolation and cDNA synthesis

Total RNA was isolated with a slightly modified CTAB-LiCl extraction method developed by Jaakola et al.^48^, as we previously described^49^. RNA samples were treated with DNase1 (ThermoFischer) according to the manufacturer’s protocol. Reverse transcription was performed from 1 µg of total RNA with random hexamers using RevertAid reverse transcriptase (ThermoFischer), as prescribed by the manufacturer. Mock reaction without RevertAid enzyme was also prepared to ensure that there is no contaminating genomic DNA in the samples.

### Quantitative real-time PCR analysis (qRT-PCR)

We selected ten core clock genes representing the three circadian loops to analyze the responses of the *Brachypodium* circadian clock to drought stress: *BdLHY1.1* (Bradi3g16515.1) and *BdLHY1.2* (Bradi3g16515.2) (this nomenclature refers to the distinction between two alternative transcript variants of the *BdLHY* gene; elsewhere in the text, *BdLHY* refers to both transcript variants), *BdTOC1* (Bradi3g48880.1), *PRR95* (Bradi4g36077.1), *BdGI* (Bradi2g05226.1), *BdLUX* (Bradi2g62067.1), *BdELF3* (Bradi2g14290.1), *ELF4-like3 *(*EFL4-3*) (Bradi4g13227.2), *ELF4-likeA *(*EFL4-A*) (Bradi4g29580.1) and *ELF4-likeB *(*EFL4-B*) (Bradi1g60090.2). *Brachypodium* core clock genes homologous to *Arabidopsis* clock components were selected based on an evolutionary analysis of clock genes by Higgins et al.^34^ and Calixto et al.^28^. Evolutionary relatedness was confirmed by reciprocal BLAST analysis and Clustal Omega alignment (https://www.ebi.ac.uk/Tools/msa/clustalo/) of *Brachypodium* and *Arabidopsis* protein sequences. For a percentage identity matrix of *Brachypodium* and *Arabidopsis* core clock genes, which summarizes putative homologues, see Supplementary Table S1. It is important to note that the *BdLHY1.2* variant is missing from the recent v3.2 *Brachypodium* Gene Models but exists in v3.1. However, we confirmed the existence of two different alternative transcripts of *BdLHY* experimentally (Supplementary Fig. S1).

Genes and related transcript sequences were collected from *Brachypodium distachyon* genome database based on the JGI v3.1 genome assembly at Phytozome v13 (https://phytozome-next.jgi.doe.gov/). Specific primer pairs for qRT-PCR detection were designed with Primer3Plus software (http://primer3plus.com/cgi-bin/dev/primer3plus.cgi) with the following parameters: 18–22 nucleotide length of primers, 110–200 bp length of products, 40–60% GC content and 60–62 °C melting temperature. Primer dimerization qualities were tested with the OligoAnalyzer Tool v.3.1 from Integrated DNA Technologies (https://eu.idtdna.com/calc/analyzer). Amplification specificities for each primer pair were checked in silico by blasting primer sequences in the NCBI PrimerBlast tool against the *B. distachyon* Refseq mRNA collection (http://www.ncbi.nlm.nih.gov/tools/primer-blast/index.cgi?LINK_LOC=BlastHome).

Primer sequences and their main attributes are listed in Supplementary Table S2. The average amplification efficiencies of each primer pair were derived from the slope of the amplification curves at the exponential phase of three different reactions from three different samples. The corresponding PCR efficiency was calculated according to the equation: E = 10(− 1/slope)^50^. We checked the selectivity of the primers towards the two BdLHY variants by sequencing the mRNA-derived PCR products (Supplementary Fig. S1).

Relative transcript amounts of the *Brachypodium* core clock genes were measured by qRT-PCR with Applied Biosystems 7900-HT Fast Real-Time detection equipment using 2 × Power UP SYBR Green PCR Master Mix (ThermoFischer) according to the manufacturer’s instructions with a standard PCR set-up: 50 °C 2 min, 95 °C 10 min, 95 °C 15 s and 60 °C 1 min at 40 cycles followed by melting point analysis. Each reaction occurred in three technical replicates and reaction specificity was confirmed by the presence of a single peak in the melting curve.

### Data analysis

Relative transcript amounts were evaluated according to the 2^−∆∆Ct^ method published by Livak and Schmittgen^51^. Results were first normalized using the average Ct values of *EF1-a* (*ELONGATION FACTOR1-a*—Bradi1g06851) and *UBC18* (*UBIQUITIN-CONJUGATING ENZYME18*—Bradi4g00660), which were validated as suitable reference genes across various plant samples^52^ and found to be appropriate for targeted analysis of time course gene expression across various light–dark and temperature cycles^44^, secondly, ∆Ct values were normalized to the average ∆Ct values for a given target clock gene of green plant parts growing at 80% water capacity at 18:6 photoperiod. All the data related to the two biological replications are available in Supplementary File 1.

All rhythm data were analyzed with Biological Rhythms Analysis Software System 2 (BRASS2), running fast Fourier transform nonlinear least-squares estimation. BRASS2 was developed and is distributed by Paul Brown and Andrew J. Millar (www.amillar.org)^53–55^. Mean periods within the circadian range (15–40 h), amplitude and relative amplitude error (RAE) values were estimated using default settings. Phase values were determined as the time of the first full peak of mRNA accumulation rhythms.

Average transcript amounts were estimated with the ∆∆Ct method for each clock gene relative to the average ∆Ct value of green plant parts growing with 80% water capacity at 18:6 photoperiod. Statistical significance of the average transcript values was predicted using a linear regression model with the R software package. Three different light conditions, two different water supplies and two different plant materials (roots or green plant parts) were used as predictors. Two different models were built for each gene: the first model (model 1) contains only the predictors while the second one also includes the interaction terms between pairs of predictors (model 2). ANOVA test was used to predict significant differences between the two models.

## Results

In order to describe the influence of mild drought stress on the circadian clock in monocots, with special attention to plant part-specific aspects, we entrained the model grass *Brachypodium distachyon* seedlings in long-day photocycles (18:6 light:dark) for two weeks. Half of the plants were then exposed to modest water deprivation (down to 40% relative soil water capacity) for two weeks before sampling. Both sets were split into three subsets: 24:0 continuous light and 0:24 continuous dark, with the third subset remaining in long-day photocycles. After exposure to the new light conditions, aerial plant parts and the total root system were collected every four hours for 3.5 days, as described in Materials and methods, resulting in a collection of 12 × 20 time-resolved samples for each of the three lighting conditions, under two different watering regimes, involving both aerial and root plant parts. The transcript quantity of the core clock genes (*LHY, TOC1, LUX, GI, PRR95, ELF3, EFL4-A, EFL4-B* and *EFL4-3*) was determined by qRT-PCR relative to the average dCT value of the green plant parts grown in 18:6 light with a normal water supply (80% relative soil water content). All the data are presented in Supplementary File 1.xlsx.

### General overview of *Brachypodium* circadian clock activity

**Figure 1:**
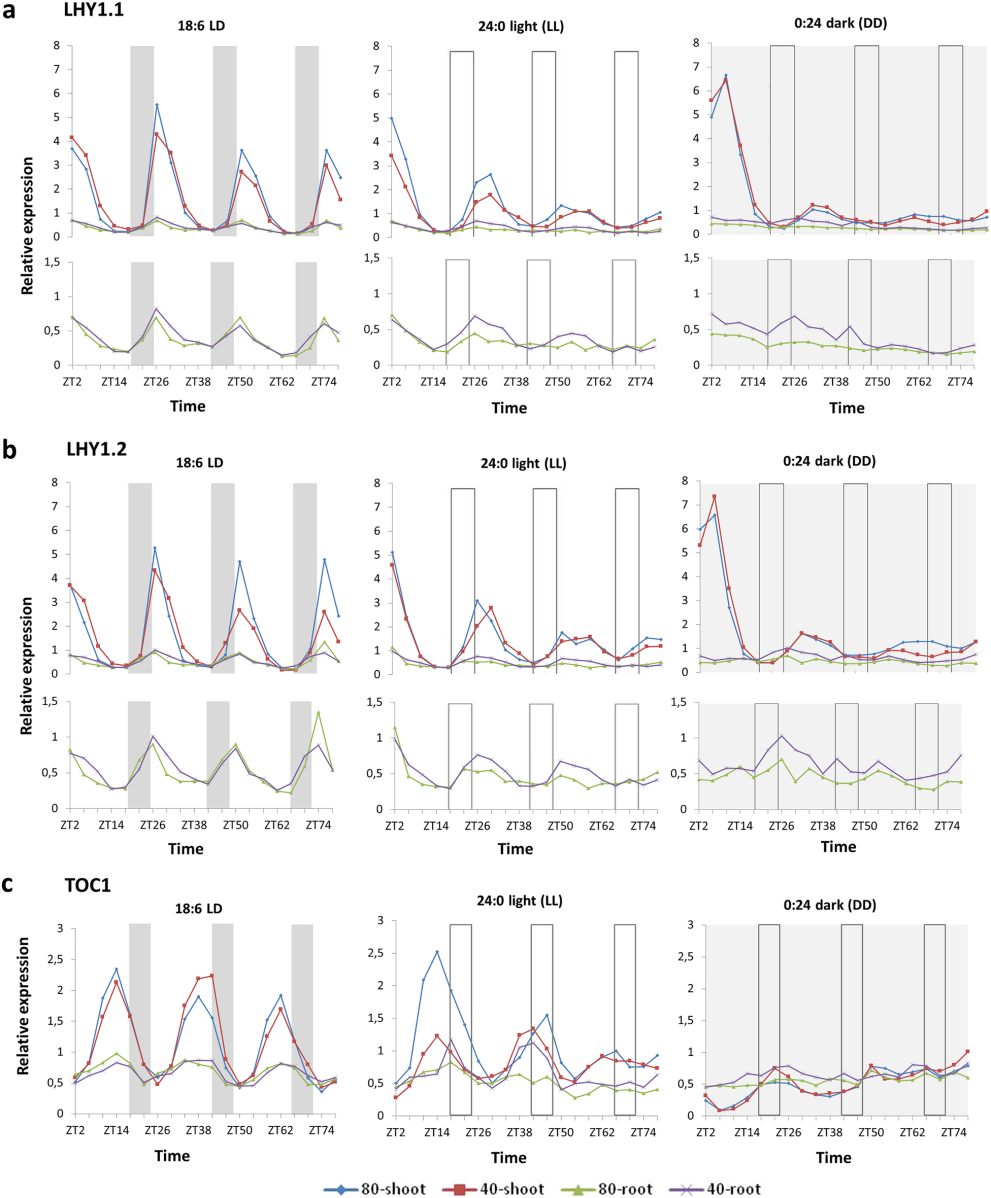
Time scale expression of *Brachypodium distachyon* central loop genes. Gene expression of two transcript variants of *BdLHY* [*BdLHY1.1* (**a**) and *BdLHY1.2* (**b**)] and *BdTOC1* (**c**) was monitored by qRT-PCR over 76 h in total aerial parts (shoot) and total roots (root) of *Brachypodium distachyon* under two watering conditions [80% soil water content (80) and 40% soil water content (40)] and three lighting regimes (18:6 light:dark, 24:0 light and 0:24 dark). Plants were entrained for 4 weeks in 18:6 light: dark cycles (light period: 6:00 to 24:00; dark period: 24:00 to 6:00) before being exposed to the three different lighting regimes. As regards water status, plants were grown with 80% soil water content for 2 weeks before being subjected to modest water deprivation (40% soil water content) for 2 weeks before sampling. Bars represent the periods of night (grey bars) and subjective night (empty bars). Expression levels shown are relative to average expression of two reference genes (*BdUBC18* and *BdElFα*) and relative to the mean expression level of the target gene over 76 h in shoots grown under 18:6 light:dark cycles and 80% soil water content. Data shown are from two representative experiments. The related period, phase, amplitude and relative amplitude error dataset can be found in Table 1. The standard deviation and related expression dataset from the two experiments are shown in Supplementary File 1.

**Figure 2:**
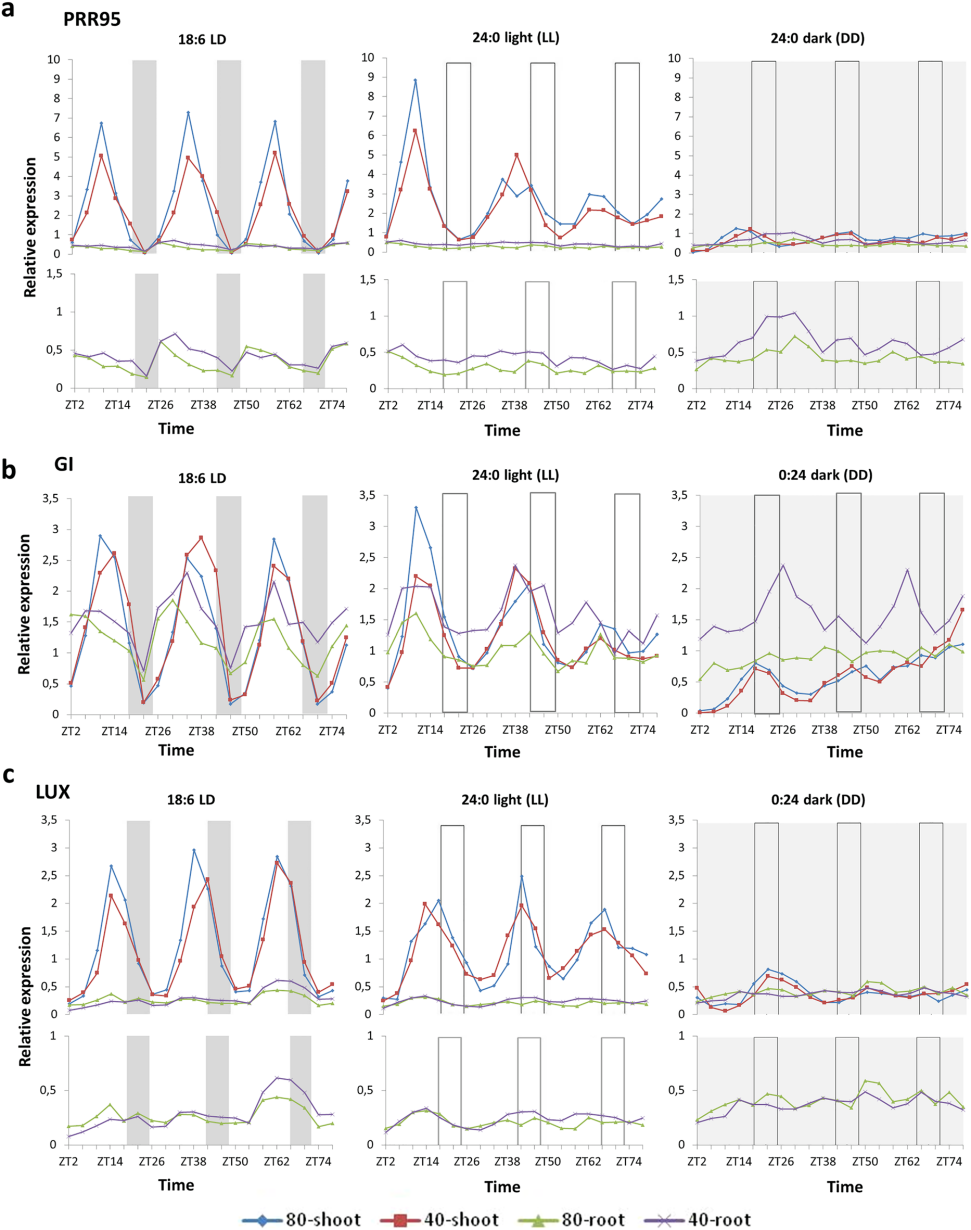
Time scale expression of *Brachypodium distachyon PRR95, GI* and *LUX* genes. Gene expression of *BdPRR95* (**a**), *BdGI* (**b**) and *BdLUX* (**c**) was monitored by qRT-PCR over 76 h in total aerial parts (shoot) and total roots (root) of *Brachypodium distachyon* under two watering conditions [80% soil water content (80) and 40% soil water content (40)] and three lighting regimes (18:6 light:dark, 24:0 light and 0:24 dark). Plants were entrained for 4 weeks in 18:6 light:dark cycles (light period: 6:00 to 24:00; dark period: 24:00 to 6:00) before being exposed to the three different lighting regimes. As regards water status, plants were grown with 80% soil water content for two weeks before being subjected to modest water deprivation (40% soil water content) for two weeks before sampling. Bars represent the periods of night (grey bars) and subjective night (empty bars). Expression levels shown are relative to the average expression of two reference genes (*BdUBC18* and *BdElFα*) and relative to mean expression level of the target gene over the 76 h in shoots grown under 18:6 light:dark cycles with 80% soil water content. Data shown are from two representative experiments. The related period, phase, amplitude and relative amplitude error dataset can be found in Table 2. Standard deviation and related expression dataset from the two experiments are shown in Supplementary File 1.

An overview of the parameters (phase, period, amplitude and RAE) under the three light conditions and two water regimes confirmed that all the clock genes investigated have rhythmic expression under long-day conditions (18:6 LD) both in the shoots and roots (Tables 1 and 2) except for *BdELF3* and *BdELF4-like* genes (Supplementary Table S3), suggesting that those genes are not components of the classical feedback regulatory loop of the *Brachypodium* central oscillator. In the presence of rhythmic entraining signal, the period of the clock genes must be parallel to the period of the signal. Not surprisingly, the period of the clock genes in LD is 24 h, which matches the period of light:dark cycles in our experiment. The expression phase of *BdLHY* in LD seems to be earlier in the roots than in the green plant parts if the plants are well-watered, but drought causes a slight phase delay (Table 1, Fig. 1a,b). Considering the standard deviation of phase estimation, these subtle differences in *BdLHY* phase are insignificant (Table 1). The expression phase of *BdTOC1* is 12 h in LD irrespective of plant parts and water conditions (Table 1, Fig. 1c). Phase of *BdPRR95* and *BdGI* expression is earlier in the roots than in shoots in LD and unaffected by water depletion (Table 2, Fig. 2). Low RAE values in the green plant parts in LD suggest a robust rhythm of the circadian clock, while the oscillation in the roots is weaker both under well-watered and drought conditions. RAE values varying between 0 (perfect fitted rhythm) and 1 (rhythm not significant) define the Fourier fit of experimental data to the algorithm-based estimated curve (the value of the amplitude error estimation divided by the experimental amplitude value)^55^. The RAE values are consistently higher, but amplitudes are lower in the drought-stressed samples. These differences in RAE and amplitude between the stressed and well-watered plants are subtle, but the tendency suggests a negative effect of drought stress on the robustness of the circadian clock except for *LHY1.2* (Tables 1, 2).

**Table 1:** Periods, phase and amplitudes and RAE values of time course gene expression of central loop genes: *LHY1.1* (Bradi3g16515.1), *LHY1.2* (Bradi3g16515.2) and *TOC1* (Bradi3g48880.1) in green plant parts and roots. Period and RAE values were estimated with BRASS software from the whole dataset (ZT2 to ZT78) for the individual time course expression curves measured by qRT-PCR. Amplitudes were calculated from the 2nd peak of the curve for *LHY1.1* and *LHY1.2*, while the 1st peak was taken into account for *TOC1*. Phase: time of expression maxima with reference to daybreak. Amplitude: twice the average distance from the mean expression maxima. RAE: relative amplitude error; Fourier fit to experimental data varying between 0 (perfect fitted rhythm) and 1 (rhythm not significant). Values are means ± SD from two biological replicates (except *, where only one dataset was appropriate for analysis). n/a: no data result from data analysis. LD: 18:6 light: dark cycles. LL: 24:0 light. DD: 0:24 dark. 80%: well-watered conditions; 80% relative soil water content. 40%: drought stress conditions; 40% relative soil water content.

				Period (h)	SD (period)	Phase (h)	SD (phase)	Amplitude	SD (amplitude)	RAE	SD (RAE)
LHY1.1	80%	Green plant parts	LD18/6	24.10	0.18	28.00	0.00	2.27	0.16	0.34	0.09
			LL	25.32	0.33	28.00	0.00	1.37	0.02	0.73	0.03
			DD	n/a	n/a	30.00	2.83	n/a	n/a	n/a	n/a
		Root	LD18/6	24.35	0.13	26.00	2.83	0.26	0.03	0.44	0.08
			LL	n/a	n/a	26.00	2.83	n/a	n/a	n/a	n/a
			DD	n/a	n/a	24.00	–*	n/a	n/a	n/a	n/a
	40%	Green plant parts	LD18/6	23.76	0.27	28.00	0.00	2.05	0.10	0.32	0.09
			LL	26.16	0.19	28.00	0.00	0.89	0.05	0.72	0.05
			DD	n/a	n/a	30.00	2.83	n/a	n/a	n/a	n/a
		Root	LD18/6	23.43	0.08	28.00	0.00	0.32	0.10	0.50	0.09
			LL	27.48	1.21	26.00	2.83	0.19	0.03	0.73	0.01
			DD	n/a	n/a	28.00	–*	n/a	n/a	n/a	n/a
LHY1.2	80%	Green plant parts	LD18/6	23.99	0.00	26.00	2.83	2.30	0.10	0.26	0.01
			LL	25.10	0.65	26.00	2.83	1.34	0.14	0.77	0.02
			DD	n/a	n/a	28.00	0.00	n/a	n/a	n/a	n/a
		Root	LD18/6	24.35	0.22	24.00	0.00	0.36	0.09	0.55	0.08
			LL	n/a	n/a	24.00	0.00	n/a	n/a	n/a	n/a
			DD	n/a	n/a	20.00	–*	n/a	n/a	n/a	n/a
	40%	Green plant parts	LD18/6	23.61	0.49	28.00	0.00	1.86	0.06	0.44	0.03
			LL	26.39	0.29	32.00	0.00	1.20	0.02	0.70	0.11
			DD	n/a	n/a	32.00	0.00	n/a	n/a	n/a	n/a
		Root	LD18/6	23.03	0.36	26.00	2.83	0.36	0.04	0.45	0.08
			LL	26.51	0.72	26.00	2.83	0.27	0.04	0.53	0.19
			DD	n/a	n/a	26.00	2.83	n/a	n/a	n/a	n/a
TOC1	80%	Green plant parts	LD18/6	23.80	0.07	12.00	0.00	1.22	0.25	0.26	0.08
			LL	27.51	0.86	12.00	0.00	0.75	0.01	0.55	0.09
			DD	30.25	1.01	20.00	0.00	0.17	0.02	0.53	0.06
		Root	LD18/6	24.17	0.27	12.00	0.00	0.25	0.02	0.45	0.06
			LL	n/a	n/a	14.00	2.83	n/a	n/a	n/a	n/a
			DD	n/a	n/a	22.00	2.83	n/a	n/a	n/a	n/a
	40%	Green plant parts	LD18/6	24.20	0.06	12.00	0.00	1.12	0.00	0.23	0.09
			LL	25.00	1.51	12.00	0.00	0.27	0.14	0.54	0.04
			DD	n/a	n/a	20.00	0.00	n/a	n/a	n/a	n/a
		Root	LD18/6	24.37	0.22	12.00	0.00	0.23	0.02	0.49	0.08
			LL	n/a	n/a	16.00	0.00	n/a	n/a	n/a	n/a
			DD	n/a	n/a	20.00	5.66	n/a	n/a	n/a	n/a

**Table 2:** Periods, phase and amplitudes and RAE values of time course gene expression of *PRR95* (Bradi4g36077), *GI* (Bradi2g05226) and *LUX* (Bradi2g62067) in green plant parts and roots. Period and RAE values were estimated with BRASS software from the whole dataset (ZT2 to ZT78) for the individual time-course expression curves measured by qRT-PCR. Phase: time of expression maxima with reference to daybreak. Amplitude: twice the average distance from the mean expression maxima. RAE: relative amplitude error; Fourier fit to experimental data varying between 0 (perfect fitted rhythm) and 1 (rhythm not significant). Values are means ± SD from two biological replicates. n/a: no data result from data analysis. LD: 18:6 light:dark cycles. LL: 24:0 light. DD: 0:24 dark. 80%: well-watered conditions; 80% relative soil water content. 40%: drought stress conditions; 40% relative soil water content.

				Period (h)	SD (period)	Phase (h)	SD (fphase)	Amplitude	SD (amplitude)	RAE	SD (RAE)
PRR95	80%	Green plant parts	LD18/6	23.67	0.06	8.00	0.00	2.73	0.31	0.27	0.02
			LL	24.96	0.74	8.00	0.00	1.21	0.42	0.84	0.11
			DD	27.16	0.08	12.00	0.00	0.27	0.05	0.77	0.01
		Root	LD18/6	24.75	0.13	4.00	0.00	0.20	0.03	0.56	0.09
			LL	n/a	n/a	6.00	1.69	n/a	n/a	n/a	n/a
			DD	n/a	n/a	2.00	0.22	n/a	n/a	n/a	n/a
	40%	Green plant parts	LD18/6	23.38	0.39	10.00	2.83	2.05	0.04	0.32	0.03
			LL	25.29	1.52	8.00	0.00	1.19	0.39	0.74	0.03
			DD	27.69	0.74	16.00	0.00	0.23	0.07	0.81	0.09
		Root	LD18/6	n/a	n/a	4.00	0.00	n/a	n/a	n/a	n/a
			LL	n/a	n/a	12.00	1.88	n/a	n/a	n/a	n/a
			DD	n/a	n/a	2.00	0.22	n/a	n/a	n/a	n/a
GI	80%	Green plant parts	LD18/6	23.85	0.05	10.00	2.83	1.37	0.10	0.23	0.05
			LL	26.57	0.42	12.00	0.00	0.73	0.02	0.74	0.13
			DD	n/a	n/a	16.00	0.00	n/a	n/a	n/a	n/a
		Root	LD18/6	24.83	0.01	4.00	0.00	0.44	0.00	0.64	0.01
			LL	n/a	n/a	n/a	n/a	n/a	n/a	n/a	n/a
			DD	n/a	n/a	n/a	n/a	n/a	n/a	n/a	n/a
	40%	Green plant parts	LD18/6	23.65	0.19	12.00	0.00	1.25	0.08	0.26	0.03
			LL	24.83	0.98	12.00	0.00	0.50	0.22	0.66	0.07
			DD	n/a	n/a	18.00	2.83	n/a	n/a	n/a	n/a
		Root	LD18/6	23.93	0.91	6.00	0.56	0.56	0.08	0.63	0.07
			LL	n/a	n/a	n/a	n/a	n/a	n/a	n/a	n/a
			DD	n/a	n/a	n/a	n/a	n/a	n/a	n/a	n/a
LUX	80%	Green plant parts	LD18/6	23.60	0.13	12.00	0.00	1.40	0.11	0.34	0.03
			LL	24.97	0.47	16.00	0.00	0.71	0.01	0.57	0.16
			DD	30.24	0.55	22.00	2.83	0.20	0.01	0.70	0.13
		Root	LD18/6	24.71	0.57	n/a	n/a	0.09	0.01	0.85	0.06
			LL	n/a	n/a	12.00	0.00	n/a	n/a	n/a	n/a
			DD	n/a	n/a	n/a	n/a	n/a	n/a	n/a	n/a
	40%	Green plant parts	LD18/6	23.59	0.11	16.00	0.00	1.12	0.03	0.39	0.06
			LL	24.69	0.68	16.00	0.00	0.56	0..19	0.56	0.05
			DD	27.48	0.35	24.00	0.00	0.19	0.00	0.71	0.04
		Root	LD18/6	24.69	0.56	n/a	n/a	0.13	0.02	0.83	0.08
			LL	n/a	n/a	10.00	2.83	n/a	n/a	n/a	n/a
			DD	n/a	n/a	n/a	n/a	n/a	n/a	n/a	n/a

### Expression of *ELF3* and *ELF4-like* genes (*EFLs*) in *Brachypodium* roots seems not to be circadian

**Figure 3:**
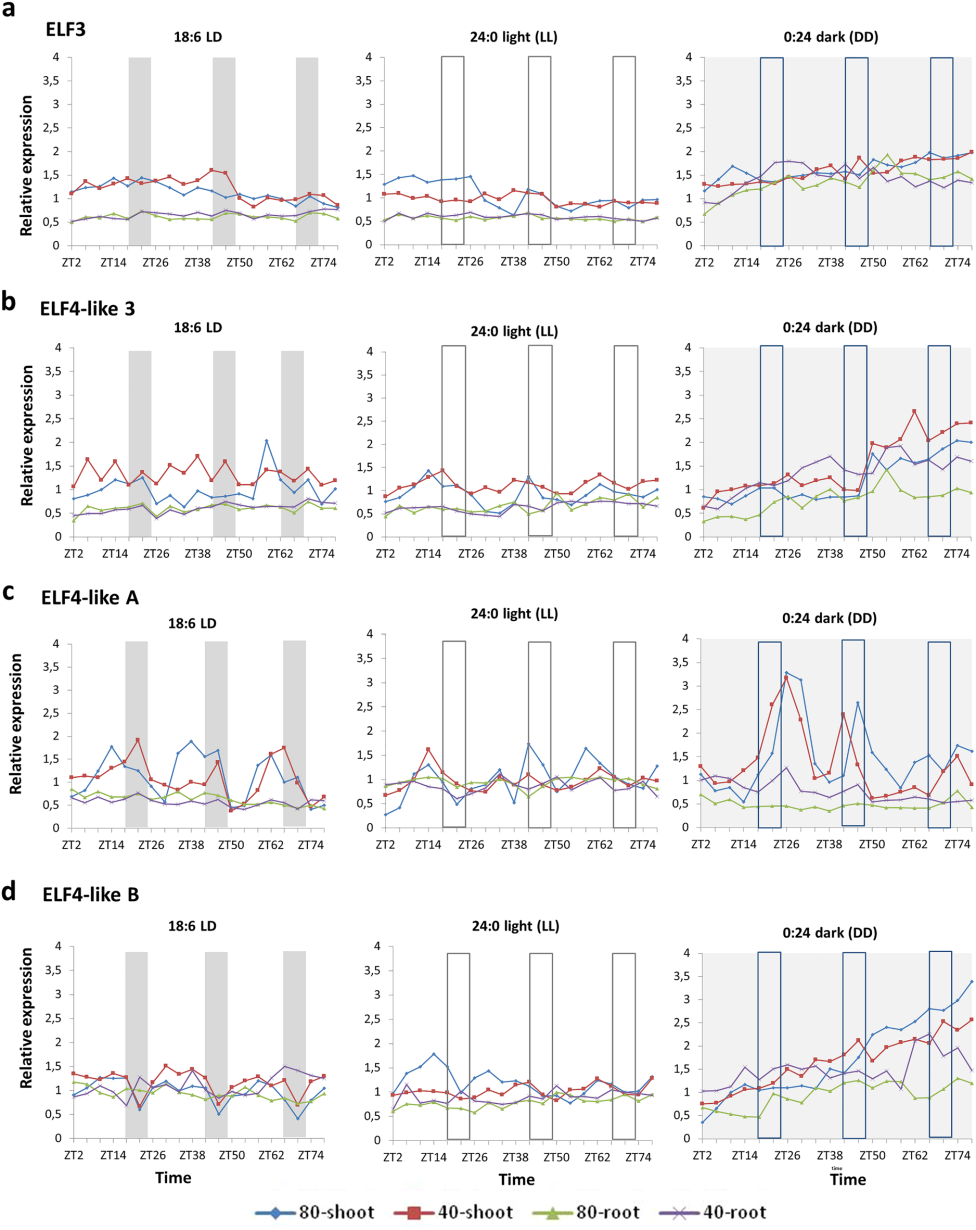
Time scale expression of *Brachypodium distachyon ELF*-related genes. Gene expression of *BdELF3* (**a**) and *BdELF4-like* genes [*ELF4-like 3* (**b**), *ELF4-like A* (**c**) and *ELF4-like B* (**d**)] were monitored by qRT-PCR over 76 h in total aerial parts (shoot) and total roots (root) of *Brachypodium distachyon* under two watering conditions [80% soil water content (80) and 40% soil water content (40)] and three lighting regimes (18:6 light:dark, 24:0 light and 0:24 dark). Plants were entrained for 4 weeks in 18:6 light:dark cycles (light period: 6:00 to 24:00; dark period: 24:00 to 6:00) before being exposed to the three different lighting regimes. As regards water status, plants were grown under 80% soil water content for 2 weeks before being subjected to modest water deprivation (40% soil water content) for 2 weeks before sampling. Bars represent the periods of night (grey bars) and subjective night (empty bars). Expression levels shown are relative to the average expression of two reference genes (*BdUBC18* and *BdElFα*) and relative to the mean expression level of the target gene over the 76 h in shoot grown under 18:6 light:dark cycles and 80% soil water content. Data shown are from two representative experiments. Related period, phase, amplitude and relative amplitude error dataset can be found in Supplementary Table S3. Standard deviation and related expression dataset from the two experiments are shown in Supplementary File 1.

In our experiment, time course expression of *BdELF3* and *BdELF4-like* genes differed markedly from that of what can be expected from classical clock genes. The *BdELF3* expression showed a steady-state expression both in the roots and green plant parts under LD, LL and DD light conditions, and there wasn’t any difference in the gene expression as regards the two watering regimes. In the green plant parts, *BdELF3* expression level was generally higher compared to the roots under LD and LL conditions, but the mean relative transcript amount for *BdELF3* in constant dark was the same in the shoots and roots (Fig. 3a). Wave parameters for *BdELF3* and *BdELF4-like* genes whit a lack of circadian regulation are presented in Supplementary Table S3.

Like *BdELF3, BdELF4-like3* showed a flattened time-course expression profile, while hardly any kind of rhythm was detected in *BdELF4-like3* transcript levels (Fig. 3b, Supplementary Table S3). Expression of *BdELF4-likeA* seems to be rhythmic either (Fig. 3c). It showed a faint oscillation in LD in the green plant parts with a peak around evening (ZT14, ZT38 and ZT62) in the well-watered samples, which is not in phase with *BdLUX* expression. The peak of *BdEFL4-like3* seems to be shifted towards the middle of the night in the green parts under drought stress (ZT22, ZT46 and ZT66) although the amplitude is lower compared to the well-watered shoots. Oscillation of *BdELF4-likeA* was not sustained in LL. Although peaks in time course expression of *BdELF4-likeA* in constant dark can be disclosed in the green plant parts, it tends not to be periodic, thus indicating that *BdELF4like-A* might be diurnally regulated in the shoots (Supplementary Table S3). Interestingly, *BdELF4-likeA* did not oscillate in the roots at all and its expression level remained unchanged irrespective of water status or light conditions (Fig. 3c).

Similar to that of *BdELF4-likeA, BdELF4-likeB* expression only oscillated slightly in LD in the green plant parts, but water depletion had no effect on it. Some faint oscillation was detected in the well-watered green plant parts under LL conditions but not in the drought-stressed ones. Like that of *BdELF4-likeA*, expression of *BdELF4-likeB* was not rhythmic in the roots under any of our experimental conditions and water depletion had no effect on it (Fig. 3d and Supplementary Table S3). Generally speaking, *ELF4-like* genes in *Brachypodium* might have a marginal role in circadian regulation as their expression profile was not circadian. Although some faint rhythm was detected in time course expression in some rare cases, these exceptions might be negligible due to the extremely low expression maxima and because the oscillation was not maintained under constant conditions (LL or DD). MacKinnon et al. also reported that without cycling external conditions (light:dark cycles or high:low temperature cycles), *Brachypodium ELF3* and *ELF4like-A* expression have no rhythm, thus leading to the assumption that temperature might have a stronger influence than photoperiod on the transcript periodicity of *BdELF*-family members^44^. Similar phenomena were reported for *ELF4-like* genes in *Arabidopsis*^39^. Similarities in expression patterns suggest that functions of *BdELF4-like* genes are closer to those of *Arabidopsis EFL4-like* genes rather than those of *AtELF4*.

### Roots vs. green plant parts

**Figure 4:**
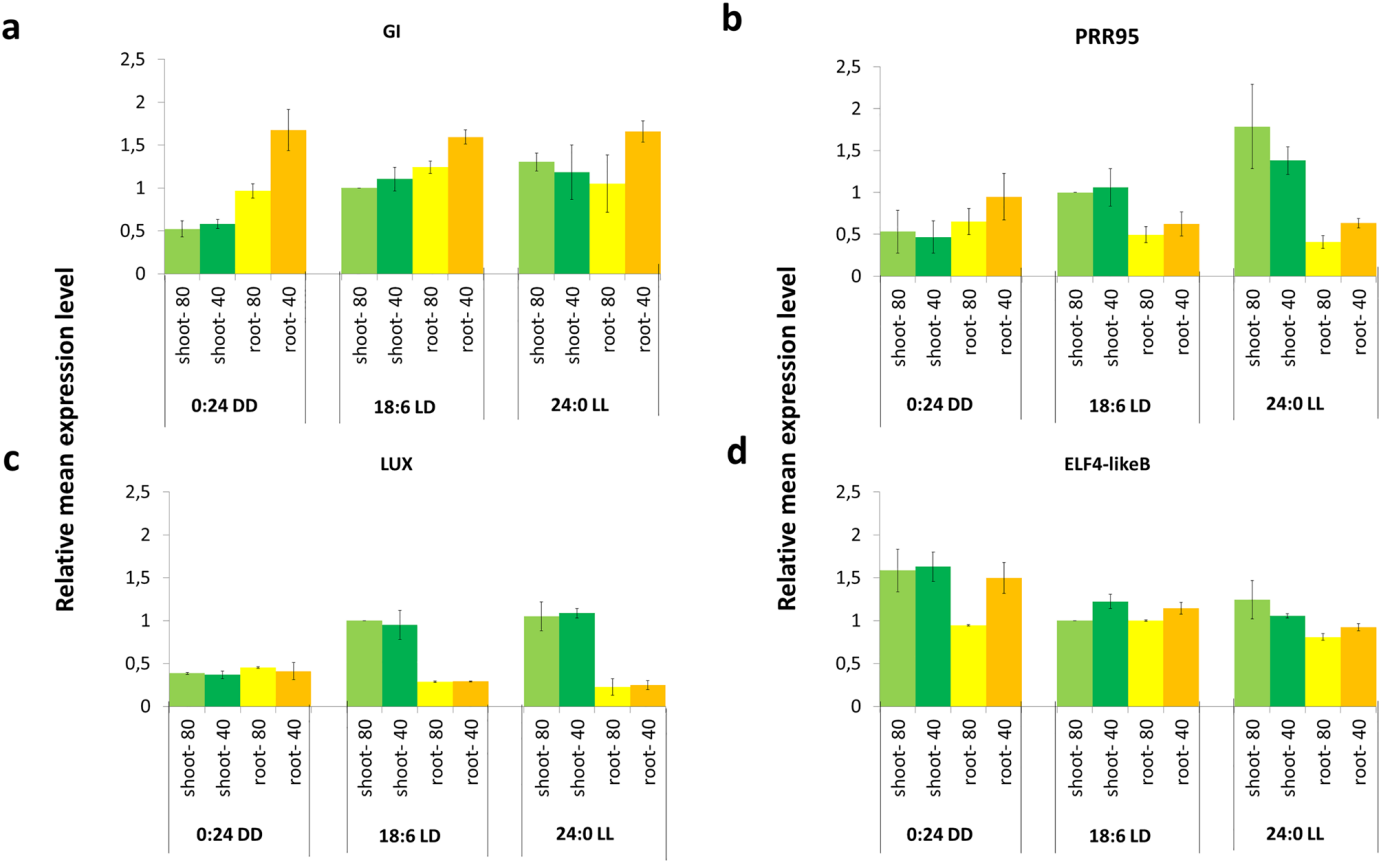
Relative mean expression of *Brachypodium GI* (**a**), *PRR95* (**b**), *LUX* (**c**) and *ELF4-like B* (**d**) genes in roots and shoots under three different lighting regime and two different kinds of soil water content. Mean transcript levels were calculated from overall relative transcript amounts of the target gene monitored by qRT-PCR every 4 h over a 76-h long time course experiment in total aerial parts (shoot) and total roots (root) of 4-week-old *Brachypodium distachyon* plants grown under two watering conditions [80% soil water content (80) and 40% soil water content (40)] and three lighting regimes [(18:6 light: dark (18:6 LD), 24:0 light (24:0 LL) and 0:24 dark (24:0 DD)]. Mean transcript levels were normalized to mean transcript level of shoots under 18:6 LD conditions and 80% soil water content. Plants were entrained for 4 weeks in 18:6 light: dark cycles before releasing them in the three different lighting regimes. As regards water status, plants were grown with 80% soil water content for 2 weeks before being subjected to modest water deprivation (40% soil water content) for 2 weeks before sampling. Error bars are ± SD for two independent experiments. Statistical analyses were performed by linear regression model and by linear regression supplemented with interacting prediction of the three different interactors: lighting regime, water status and plant part. Statistical testing of the models is summarized in Table 3. Values are statistically different (p < 0.05 by ANOVA) if interaction between the parameters is predicted. This figure presents genes with significant interaction. Relative mean expression values of *BdLHY1.1, BdLHY1.2, TOC1, ELF3, ELF4-like 3* and *ELF4-like A* can be found in Supplementary Fig. S2.

In order to test the simultaneous effect of the three parameters (light conditions, watering conditions, and aerial parts vs. roots) on the average expression level of clock genes, we used two different statistical models; a linear regression and a linear regression combined with interacting parameters. Statistical test of the two models demonstrated significant interaction of the parameters for four clock genes: *BdGI, BdPRR95, BdLUX* and *BdELF4-like B* (Fig. 4 and Table 3). Comparison of mean expression levels showed that the mean expression levels of the core clock genes in the roots are significantly lower than in the green plant parts in most cases—such as *BdPRR95* and *BdLUX* (Fig. 4b,c); consistent with low robustness. The only exception is *BdGI*, whose average expression level in the roots exceeds the values of the green plant parts (Fig. 4a). Moreover, water depletion elevated only the mean expression level of *BdGI* in the roots, especially in constant dark. The known role of *Arabidopsis* GI in drought tolerance and interplay between GI and ABA signaling^56,57^ might explain the rising effect of water depletion on the mean expression level of *BdGI*. However, we only observed this phenomenon in the roots, while *BdGI* responded to water depletion in green plant parts with a slight phase delay, thus suggesting that drought stress affects *BdGI* expression differently in different organs. Nevertheless, the mean expression levels of *Brachypodium* core clock genes in the roots are similar under all of the tested light conditions. In contrast, the mean expression levels of most clock genes—such as *BdPRR95* and *BdLUX*—were elevated by light in the green plant parts. For example, the mean expression level of *BdPRR95* is two times higher in LD and four times higher in LL compared to DD as regards the green plant parts (Fig. 4b). Irregular behavior was only found for *BdELF* family members (*BdELF3* and *BdELF4-like* genes) (Fig. 4d).

**Table 3:** Statistical test of the effect of three parameters (lighting regime, water status and plant organ) on the mean relative transcript amount of *Brachypodium* core clock genes. Model 1: linear model; Model 2: linear model with interacting factor. R-squared means the coefficient determination that indicates the fitting quality of the model. Values are statistically different (p < 0.05 by T-test ANOVA) if interaction between the parameters is predicted. p < 0.05 indicates a significant difference between the two models.

	Model 1 R-squared	Model 2 R-squared	p-value	
LUX	0.63628	0.96912	0.00002	***
PRR95	0.38018	0.87195	0.00239	**
GI	0.61655	0.89894	0.00884	**
ELF4-2/ELF4-like B	0.66267	0.89569	0.02028	*
ELF4-1/ELF4-like 3	0.68375	0.84664	0.17265	
TOC1	0.27147	0.51893	0.54821	
ELF4-3/ELF4-like A	0.37674	0.55759	0.67213	
LHY1.1	0.75202	0.78863	0.94206	
LHY1.2	0.75793	0.79321	0.94416	
ELF3	0.67610	0.69413	0.99721	

The mean expression levels of the *BdELF* genes (including the *BdELF3* and *ELF4-like* genes) were the highest in constant dark, and they decreased whit light input. As regards the *BdELF4-like* genes, the relative transcript amounts were similar under all the conditions and plant parts tested except for *BdELF4-like B*, whose mean expression level was elevated by water depletion in the roots in DD (Fig. 4d). Considering the high mean expression levels of *BdELF*-family genes in DD, it is possible that we could not observe rhythmic time course expression in LD as regards *BdELFs* due to long-day growing conditions and short nights. Reconciling the controversial time course expression profiles and relatively low mean expression levels of *ELFs* in *Brachypodium* with their evolutionary history, it is entirely possible that their functions differ from those of *Arabidopsis ELF3* and *ELF4*. See Supplementary Figure S2 for results referring to the whole sets of core clock genes.

### Period of *BdPRR95* expression differs in the roots from that in the shoots in LD

The *Arabidopsis* PSEUDO-RESPONSE REGULATORS function sequentially as transcriptional repressors throughout the day in the following order: PRR9 (morning), PRR7 and PRR5 (afternoon), PRR3 (before evening) and TOC1/PRR1 (evening)^3^. For our time course expression analysis experiment to represent *Brachypodium* PRR family, *BdPRR95* was selected since it has the less ambiguously predicted genomic environment and the fewest alternative transcript variants among PRRs in the *Brachypodium* genome database.

*BdPPR95* in LD shows an oscillating expression profile with a sharp peak in the afternoon (ZT10, ZT34 and ZT58) in the shoots, while this peak appears around midday in the roots (ZT6, ZT30, ZT54 and ZT78) (Fig. 2a). *BdPRR95* was scored as rhythmic both in the green plant parts and roots in LD, but the oscillation phase differed in the roots from that in the aerial parts. In the roots oscillation is weak, and amplitude is ten times lower relative to the green plant parts (Table 2). Water depletion has no effect on *BdPRR95* expression either in the green plant parts nor in the roots in LD. Transcript profiles for the well-watered and stressed samples were in phase both in the green plant parts and the roots under LD conditions. In the shoots in LL, the *BdPRR95* transcript level oscillated for three full cycles with a peak shifted to the afternoon (ZT10, ZT38 and ZT62), and this pattern was unaffected by water depletion. Some dampening was observed in the amplitude (Fig. 2a). In contrast to the green plant parts, *BdPRR95* did not oscillate in the roots under LL conditions. However, the roots had a slightly elevated relative transcript amount under drought stress. In DD, *BdPRR95* rhythm is less robust (the amplitude is smaller), and the phase was shifted to the beginning of subjective night (ZT14 and ZT46) in the green plant parts (Fig. 2a, Table 2). However, *BdPRR95* expression continued oscillating in the shoots in DD for two more cycles irrespective of whether the plants were exposed to drought stress or not. In contrast, rhythm of *BdPRR95* expression was not detected in the roots in DD either under well-watered or stressed conditions (Fig. 2a).

As far as the evening-phased clock genes are concerned, differences in clock gene expression between the green plant parts and roots are more pronounced compared to the morning-phased ones (Fig. 2). This suggests that transmission of a potential light-driven signal from shoots might be responsible for entraining the circadian clock in the roots and that the evening loop might work decoupled from the morning-phased clock genes in the roots. However, if the plants were exposed to drought stress, most of the clock genes preserved their rhythmic expression in the roots in continuous light. In general, clock genes seem to be more responsive to drought in the roots than in the green plant parts, as water depletion increased the amplitude or relative transcript amounts of many clock genes in the roots but not in the green plant parts.

### Maintenance of circadian rhythm under constant light.

The phase of *BdTOC1* expression pattern was opposite that of the *BdLHY* transcripts in consistent with the reciprocal regulation between TOC1 and LHY (Fig. 1c). Under physiological conditions (80% LD), the *BdTOC1* expression peak was also in the middle of the evening (ZT14, ZT38 and ZT62) in the shoots and roots, and water depletion did not affect this rhythm. Although there was no difference between the green plant parts and roots in LD as regards the rhythm of *BdTOC1* expression, the relative amplitude of oscillation was significantly higher in the green plant parts (Table 1). The effect of water depletion on *BdTOC1* expression appeared under LL conditions. Oscillation of *BdTOC1* expression was visible in shoots of the well-watered plants for three full cycles in constant light with some dampening, and the peak of expression was shifted towards the very end of subjective day (Fig. 1c). This dampening and phase shifting was less robust in the stressed green plant parts in LL. In roots of the well-watered plants, *BdTOC1* expression rhythm disappeared quickly in LL. In contrast, *BdTOC1* transcript profile preserved its free running rhythm for two more cycles in LL under drought stress in the roots (Fig. 1c). In green plant parts under DD, *BdTOC1* showed a slightly rising expression with subtle oscillation, and there was no difference between the well-watered and stressed samples (Table 1). In the roots, *BdTOC1* oscillation flattened rapidly with little variation between the well-watered and drought samples. As water depletion markedly affected *BdTOC1* expression only in roots under LL, it might be assumed that there is an organ-specific effect of drought on *BdTOC1* that depends on light (Fig. 1c).

The light intensity-dependent free running period of the circadian clock can be monitored in LL. Without the entraining signal of light:dark cycles the amplitude and RAE values of the *Brachypodium* circadian clock genes in LL are lower compared to LD, which is in consistent with general knowledge about the plant circadian clock^7^. In LL, *BdTOC1* period was not detected in roots thus indicating a dampened clock function in that organ. In green plant parts, *BdTOC1* transcript profile has a slightly longer period compared to that of *BdLHY* (25 h for *BdLHY* and 27 h for *BdTOC1*). However, in response to water deprivation, *BdTOC1* transcript level and period decreased while that of *BdLHY* increased in the shoots, thus equalizing the difference in the period length between *BdTOC1* and *BdLHY* (Table 1). A longer period was observed for all the clock genes (apart from *BdELF3* and *ELF4-like* genes), suggesting that the internal period for *Brachypodium* might be longer than 24 h, consistent with MacKinnon and coworkers’ observations^44^. Period of *BdGI* expression in LL is significantly longer compared to other clock genes in well-watered plants, but water deprivation dampens this difference in period length between LL and LD. Some similar effects appeared for the free running period of *BdTOC1*, but drought stress has no effect on the period of *BdPRR95* and *LUX* in LL. The phase values in LL are almost the same as those in LD with the difference that we also observed changes in phase of *BdTOC1* expression in LL, in relation to the plant parts tested. It is common but not a principal that the longer period in LL is accompanied by phase delay. A modest phase-delay can be observed in the roots in the case of *BdTOC1*, while phase of *BdLHY* is earlier in the roots compared to the green plant parts (Table 1). Notwithstanding that both *BdLHY1.1* and *BdLHY1.2* responded to water depletion with a delayed expression peak, the phase delay caused by drought was only observed in LL in the case of *BdLHY1.2*. In parallel to this, considerable increasing in period as a consequence of drought stress appeared only in the case of *BdLHY1.2*.

In LD, there was little difference between the green plant parts and roots as regards the phase and period (Table 1). Nevertheless, rhythm in the roots was less robust. Expression of the core clock genes was synchronous in the roots to that of green plant parts in LD, but the relative transcript levels and amplitude of clock gene oscillation were remarkably lower in the roots. Differences between the shoots and roots in terms of rhythm of clock gene expression appeared in LL. In continuous light, most of the examined *Brachypodium* clock genes examined failed to carry on oscillating in roots if the plants were well watered. However, the oscillation of *BdLHY* in LL continued for two more cycles in the drought-stressed roots but not in the well-watered ones (Fig. 1a,b).

There were no differences between the expression pattern of the two *BdLHY* transcript variants as regards the phase and period (Fig. 1a,b and Table 1). In *Arabidopsis,* alternative splicing of the putative equivalent of CCA1 is mediated by stress conditions. Seo et al. demonstrated that alternative splicing of *CCA1* is suppressed by cold, thus releasing the functional CCA1α from the competitive inhibition by CCA1β and promoting freezing tolerance in *Arabidopsis*^37^. Moreover, alternative splicing is supposed to be the “missing link” between the circadian clock and environmental stress adaptation in plants based on experiments which reported altered alternative splicing patterns of clock genes in *Arabidopsis* in response to changed photoperiod, temperature extremes and salt stress^58^. By comparing the average transcript levels of *BdLHY1.2* and *BdLHY1.1*, we saw the same ratio in the well-watered and drought-stressed samples (Supplementary Fig. S1c). This indicates that it is the photoperiod, not water depletion that has an effect on the average transcript amount of *BdLHY1.1* and *BdLHY1.2*. When plants were exposed to light, the average transcript amount of *BdLHY1.1* was approximately three times higher in the green plant parts than that of *BdLHY1.2* irrespective of watering status, thus confirming that *BdLHY1.1* is the main transcript variant. However, there was no difference in the average transcript amounts of *BdLHY* transcript variants in the roots.

### Maintenance of circadian rhythm under constant darkness

In DD, most of the clock parameters cannot be identified except for the phase due to the weakness and low amplitude of the circadian clock. Even estimation of phases is error-prone in constant darkness, as indicated by the high SD values. Nevertheless, the clock phase in DD is delayed in comparison to LD or LL, implying a relatively long clock period in constant dark—significantly longer than that of the circumstances when the circadian clock gets light input, similarly to *Arabidopsis*^27^ (Figs. 1, 2, Tables 1, 2).

### Members of the central loop respond differently to water depletion in roots compared to the green plant parts under continuous light

Under long-day conditions, both transcript variants of *BdLHY* show oscillating expression patterns. the rhythm of the oscillation is the same in the roots and green plant parts with a peak at dawn (ZT2, ZT26, ZT50 and ZT74)—immediately after the set time point of dawn, which is essentially the same as those described for LHY and CCA1 in *Arabidopsis*^59^ (Fig. 1a,b). Although there is no significant difference between the green plant parts and roots as regards the phase of *BdLHY* expression, the relative transcript level and amplitude are significantly lower in the roots (Table 1). Relative transcript amount of both *BdLHY* transcript variants at the peaks of expression is approximately six times higher in the shoots than in the roots, independently of water status. Water depletion seems to only have a subtle effect on *BdLHY* expression in LD. However, under drought conditions, both transcript variants of *BdLHY* have lower amplitude compared to the well-watered samples in green plant parts, while the amplitude of the oscillation remained at the level of the well-watered samples or even a bit higher in the stressed roots (Table 1). The difference between roots and green plant parts in response to water limitation as regards the *BdLHY* expression is more obvious in LL (Fig. 1a,b). One of the most important criteria for proving circadian regulation is the maintenance of rhythmic expression for a while in the absence of entraining signal—such as periodic lighting—under constant conditions with a period of about 24 h (a so-called free running rhythm)^1^. In the green plant parts, *BdLHY* continues oscillating in LL with decreasing amplitude and a subtle shift in its period to ~ 26 h. The free running rhythm of *BdLHY* expression in LL was independent of watering conditions in the green plant parts. However, oscillating expression of *BdLHY* in the roots in LL was only maintained in response to water depletion for a longer period (~ 27 h) compared to the green plant parts (Table 1). Oscillating expression of *BdLHY* was not detectable in the well-watered root samples after 24 h of continuous light.

In order to test if this phenomenon is related entirely to water depletion or might be light-driven, we also measured *BdLHY1.1* and *BdLHY1.2* expression during three-and-a half days of constant darkness (DD). Rhythmic expression of both *BdLHY* transcript variants continued in darkness at a lower amplitude, and a two-hour phase shift appeared in the shoots. Drought stress had no visible impact on *BdLHY* expression in DD in the green plant parts. Interestingly, the oscillation of *BdLHY* expression was not detectable either in the stressed roots or in the well-watered ones in DD even though both *BdLHY* transcripts showed an elevated relative transcript amount in drought. This suggests that the oscillation of *BdLHY* in roots depends on light, but water depletion enhances it (Fig. 1a,b).

### Water depletion acts differently on GI and LUX expression

Evening loop components [GI, LUX and EARLY FLOWERING genes (ELF3 and ELF4)] provide a direct link for the central loop towards the pleiotropic outputs of the circadian system. As they form a common regulation platform, their expression patterns are strictly overlapping with an expression maximum around the beginning of subjective night^5^.

Consonant to the expectations, the peak of *BdGI* expression is in the afternoon (ZT10, ZT34 and ZT58) in the well-watered shoots in LD. However, the expression peak of *BdGI* shifted by 4 h (ZT14, ZT48 and ZT58-62) in the green plant parts under LD in response to water depletion. The shape of the time course expression curve of *BdGI* in the roots was less robust compared to the green plant parts but oscillating under LD conditions. Intriguingly, the phase of *BdGI* expression in the shoots seemed to be delayed relative to its rhythm in the roots. In the well-watered roots in LD, expression peak of *BdGI* appeared 4 h earlier compared to the well-watered shoots (ZT6, ZT30 and ZT54). In the drought-stressed roots, this peak was shifted to the afternoon (ZT10, ZT34 and Z58) in LD (Fig. 2b). Water limitation had no influence on the relative quantity of the *BdGI* transcripts in the green plant parts. However, in the stressed roots, *BdGI* had a slightly elevated relative transcript amount in comparison to the well-watered roots. In LL and DD, expression pattern of *BdGI* oscillates for three further cycles with dampening amplitude in the shoots. Water status affected neither the free running phase nor the amplitude of *BdGI* expression in the shoots under LL and DD, although the average transcript level was significantly lower in DD compared to LL. In the well-watered roots, *BdGI* shows a long-drawn and slightly rhythmic expression in LL and does not oscillate at all in DD (Table 2). Under limited water conditions, *BdGI* had no free running rhythm in the roots either in LL or in DD, but the average expression level was higher compared to the well-watered conditions (Fig. 2b).

*BdLUX* expression was in phase with the expression of *BdGI* in the green plant parts under LD, LL and DD conditions, but phase shifting in response to drought stress was not observable (Fig. 1c). In point of fact, water depletion had no visible effect on *BdLUX* expression in the shoots in any of the lighting conditions (Table 2). Compared to the green plant parts, oscillation of *BdLUX* transcripts in the roots was not detectable under any of the conditions except for a very slight oscillation under drought stress in LL, suggesting that intensive loss of water in LL might promote cyclic expression of *LUX* in *Brachypodium* roots (Fig. 2c).

## Discussion

Timing of biological processes to the daily rotation of the Earth with an endogenous oscillator provides a highly adaptive evolutionary advantage for all living organisms. Plants possess an endogenous circadian clock consisting of plant-specific elements for temporal regulation of vital processes, for example, photosynthesis, stomatal movements, stem elongation, flowering, hormone responses, stress tolerance, and so on. Studies of species other than *Arabidopsis,* such as cereals (rice, barley and wheat), have shown the high conservation of the plant circadian clock but have simultaneously highlighted some fundamental differences^60^. Experiments on the circadian clock in agriculturally important plants have gained increasing popularity, since the circadian clock contributes greatly to grain yield and stress tolerance^61^.

In our study, we aimed to provide a comprehensive picture of the clock gene expression of a monocot model *Brachypodium distachyon* with a special emphasis on roots and drought response. Monitoring the relative transcript amount of core clock genes in green plant parts demonstrated a high correspondence in their rhythm to that of their *Arabidopis* counterparts with the exception of ELF3 and ELF4-like genes. In *Arabidopsis,* the expression of *AtELF3* and *AtELF4* is regulated rhythmically with a peak at dusk. Forming the evening protein complex in common with LUX, both ELF3 and ELF4 are required for sustaining endogenous rhythms in the absence of light/dark cycles, which have a crucial function in the circadian gating of growth promoting transcription factors and in setting flowering time, among others^62–64^. Knowing the central role of ELFs, it is surprising that ELF family members seem the most controversial in monocots in terms of the evolutionary history and functional homology. For example, a key structural difference is the absence of a prion-like domain in *Brachypodium* ELF3. This domain is responsible for reversible inactivation of ELF3 at high temperature in *Arabidopsis,* but its sequence was not predicted in *Brachypodium,* indicating a role for BdELF3 which is distinct from its *Arabidopsis* counterpart^65^. Despite structural differences, BdELF3 was able to restore the function of AtELF3 in hypocotyl elongation, clock rhythm and flowering in cross-species complementation experiments, thus indicating a conserved role of ELF3 across the monocot/eudicot lineage^43^. The role of BdELF3 in regulating clock gene expression, photoperiod sensing and flower induction was also confirmed within *Brachypodium* Bd21-3 accession, providing further evidence for functional conservancy^66^. However, at a high temperature (27 °C), *BdELF3* was unable to complement the thermally responsive early flowering phenotype of *Arabidopsis elf3* mutants^65^. As regards *ELF4-like* genes, there are no obvious orthologues of *Arabidopsis ELF4* in *Brachypodium* (or other grasses), but putative counterparts (the *ELF4-like* genes) have not yet been functionally characterized. Detailed characterization of *Brachypodium ELF-like* family genes was not among our objectives, but examining their time course expression profile under different light conditions might shed light on functional similarities and differences. However, this does not necessarily mean that *BdELF3* and *BdELF4-like* genes have no effect on the circadian clock at all (consider the complementary ability of *BdELF3* of the clock function of *Arabidopsis elf3* mutants^66^). Most of the *Brachypodium* clock components show high sequence similarity to *Arabidopsis* clock proteins. On the other hand, behavior of the *Brachypodium* clock genes in shoots has previously been reported as being very similar to that of *Arabidopsis*^44,45,67,68^. Despite differences in plant growing conditions, our experimental data are entirely consistent with the studies noted above; leading us to the conviction that the *Brachypodium* circadian clock works on a conserved manner.

Based on the estimated clock parameters, we can conclude that behavior of the *Brachypodium* circadian clock follows the general principles of plant circadian mechanisms. Robustness is significantly greater in green plant parts than in roots. Water depletion affects both the period and phase of the circadian clock, mainly in the roots. However, the strength and direction of changes in period and amplitude caused by drought differ among clock genes. This implies that feedback regulation between clock loops loses its strength and is slightly decoupled if water supply is limited. It is worth noting that a low number of biological replications (n = 2) makes the estimation of clock parameters ambiguous.

To sum up, it can be stated that the behavior of *Brachypodium* clock genes in green plant parts meets the expectations based on the known expression profiles of their *Arabidopsis* counterparts. This strengthens the generally accepted concept of functional conservation of clock genes among wide ranges of plant species. Generally speaking, the period of clock gene expression was persistent with the lack of entraining light: dark signals in *Brachypodium*, and it was longer in constant dark (DD) compared to continuous light (LL) in the green plant parts in accordance with Aschoff’s rule^69^. A dampening of amplitude is clearly visible in DD, consistent with Dalchau and coworkers’ report^70^. Water depletion had a negligible effect on clock gene expression in the green plant parts.

Previously, it was widely assumed that the circadian clock functions uniformly throughout the plant^71^. However, the concept of a generalized plant circadian clock has been reconsidered owing to experiments on tissue-specific aspects of the circadian clock, which reported that the clock can be sensitive to different cues, runs at different speeds, and drives distinct processes in different cell types, thus providing flexibility for regulating such a range of developmental and physiological processes as a master conductor^20,22,72^. Tissue specificity of the circadian clock is mainly concluded on the basis of experimenting with different green plant parts and tissues. Differences in expression patterns of clock genes in roots and shoots were revealed in *Arabidopsis* in detail, indicating a different operation of the circadian clock in roots^25,27^. James et al.^27^ reported that the period of clock gene expression in *Arabidopsis* roots in LD was exactly in phase with the shoots except for *ELF3*, which oscillated only in the shoots. However, the phase of clock gene expression was longer in *Arabidopsis* roots than that in the shoots in constant light, although the organs were synchronized in LD and no evidence was found for rhythmic expression of evening-phased clock genes in the roots in LL. They showed that the period of *LHY1, CCA1, PRR7* and *PRR9* expression shifted by two hours in *Arabidopsis* roots in constant light, while the transcripts of *TOC1, GI, LUX, PRR3, PRR5, ELF3* and *ELF4* only oscillated in the shoots. In the shoots, period of clock gene expression is usually longer in DD than in LL. However, James et al.^27^ found that periods in LL and DD were similar in the roots although lower amplitude in dark-grown roots made rhythms more difficult to be detected. Based on these observations, they concluded that the morning-phased loop of the clock is in operation but that the genes in the central and evening-phased loops are decoupled from *CCA1* and *LHY* expression in *Arabidopsis* roots. They assumed that the clock in the shoots and roots has similar compositions but different dynamic properties. The circadian clock in *Arabidopsis* roots might thus be a simplified slave version of the clock in the shoots. It is more than likely that the evening complex (EC) is responsible for the differences in circadian rhythm in *Arabidopsis* shoots and roots through different sensitivity to environmental inputs^73^.

Experimental conditions we used differ from those of James et al.^27^, but our results are consistent with theirs. The behavior of core clock genes in mature *Brachypodium* roots at the level of the whole organ was similar to that of *Arabidopsis* suggesting that the concept of a simplified root clock can also be applied to *Brachypodium* and strengthening the hypothesis that the plant circadian clock might be organ-specific but not organ-autonomous. There have previously been no comprehensive reports on clock genes in monocot roots. Our results indicate that the circadian clock might act similarly in monocot and dicot roots in spite of major differences both in the architecture and structure of their root systems^74^.

Entraining signal for the circadian clock in roots is under debate. Takahashi et al.^24^ suppose that the clock in shoot apex cells synchronizes the circadian rhythm of roots via direct intercellular communication, which makes the root rhythm dampen rapidly after excision of the apex. James et al.^27^ reasoned that this shoot-driven entraining signal might be related to shoot photosynthetic metabolism and that the signal is transmitted between the organs under LD conditions. Sucrose or a derivative is proposed as a photosynthesis-related signal for entraining the circadian clock in roots^27^, but it has also been demonstrated that roots can perceive very low-intensity red light via phytocrhome B, which is capable of directly setting the circadian rhythm of the roots^25,73,75^. Moreover, the root clock seems to be entrained by light in preference to shoot-derived signals^76^. On the other hand, most recent studies have demonstrated that ELF4 is transported from the shoots to the roots in *Arabidopsis*, thus regulating the rhythm of the circadian clock in the roots in a temperature-dependent manner^77^. The role of the evening complex in direct integration of environmental signals—particularly temperature cues—into rhythmic endogenous gene expression programs is known in *Arabidopsis* shoots^78^, and trafficking of ELF4 from aerial parts to the roots might have a crucial function in delivering temperature information from the shoots towards the circadian clock in the roots. Investigating the courier function of ELF4 between shoots and roots in relation to drought stress would be interesting. Moreover, a clock component with the same messenger role in monocots would be worth identifying as monocot plants lack the true homologue of *AtELF4*.

It was far beyond our scope to identify the entraining signal for root circadian rhythm. We made an effort to reveal how water limitation modulates the circadian clock in roots. Effects of the time of day on the *Arabidopsis* growth dynamic and transcriptome in response to drought are evident, but mild drought stress has no reported effects on the expression of core clock genes^17,18^. However, these observations refer to rosette leaves, and there are no experimental data on roots. Here we demonstrate that water depletion has a noticable effect on the circadian clock in roots but not in green plant parts. Under LD conditions, the rhythm of clock genes in the drought-stressed root samples did not differ from the well-watered ones; however, in constant light, many core clock genes, such as *BdLHY, BdGI* and *BdLUX*, showed elevated relative transcript amounts and preserved their oscillating expression in the roots under drought conditions. Among core clock genes, *BdTOC1* expression was mostly affected by water depletion in the roots in LL in its expression level but not in its phase. Namely, free running oscillation of *BdTOC1* was only visible in the roots in response to water depletion under continuous light. In contrast, water depletion had little effect on the free running period of *BdTOC1* in the green plant parts, thus indicating an organ-specific effect of drought on the circadian clock. Notwithstanding that *BdTOC1* expression was less affected by drought in the green plant parts, sensitivity of *BdTOC1* to water depletion implies a role in drought stress responses. In *Arabidopsis* TOC1 has been reported as a molecular linkage between circadian clock and drought responses, since TOC1 has a confirmed reciprocal relation to ABA signaling^79,80^.

These observations raise the question of whether water status can serve as an entraining environmental signal for the circadian clock in the roots or vice versa. Oscillation of hydraulic conductance and circadian regulation of water dynamics via rhythmic expression of aquaporins in *Arabidopsis* roots suggests reciprocal regulation between water status and circadian regulation^81,82^. In addition, the rhythm of hydraulic conductance and growth was enhanced by drought in *Arabidopsis*. Dynamic regulation of water status by the circadian clock in shoots is important in toleration of water deprivation. However, the contribution of the circadian regulation of water status in roots to drought adaptation is still unknown. Moreover, biological relevance of circadian oscillations in roots has only started to be explored. Rust et al.^83^ reported that *AtCCA1* overexpression caused strong repression of lateral root formation and unusual changes in growth direction of lateral roots accompanied by increased lateral extension of the root architecture in LD. They observed the same aberrations in the *Arabidopsis prr975* triple mutant. According to recent studies, proliferation of root cells is mediated by PRR5,7,9 through repression of a zinc-finger protein central to root development^84^. Recent reports have demonstrated a resetting of the circadian clock during lateral root formation and confirmed oscillation of core clock genes in lateral root primordia^85^. Furthermore, GI controls auxin level and expression of auxin-related genes and is a positive regulator of the central pathway for lateral root initiation (the so-called “IAA14-ARF7-LBD16” module)^86^.

Periodically marking the time and place of a set of xylem pericycle cells to be selected for priming lateral root emergence by oscillating gene expression in the region close to the primary root tip pointed to the dynamic and rhythmic nature of lateral root formation^87^. Identifying the connection of this biological clock termed as a “root clock” to the circadian clock might shed light on the direct contribution of the circadian clock to root development. It might be interesting to investigate the relation of drought stress-induced developmental changes in roots to the connection between the circadian clock and the “root clock”. However, these are some of the unexplored areas of circadian clock and root development research.

## Supplementary Information

Supplementary Information 1.

Supplementary Information 2.

Supplementary Information 3.

## Data Availability

All related data are available in the form of electronic supplementary materials (Supplementary File 1).
