# Supplementary figures and images for: Limited water stress modulates expression of circadian clock genes in Brachypodium distachyon roots
Source: Sci Rep. 2023 Jan 23;13:1241. doi: 10.1038/s41598-022-27287-4 (PMC9870971; doi:10.1038/s41598-022-27287-4)

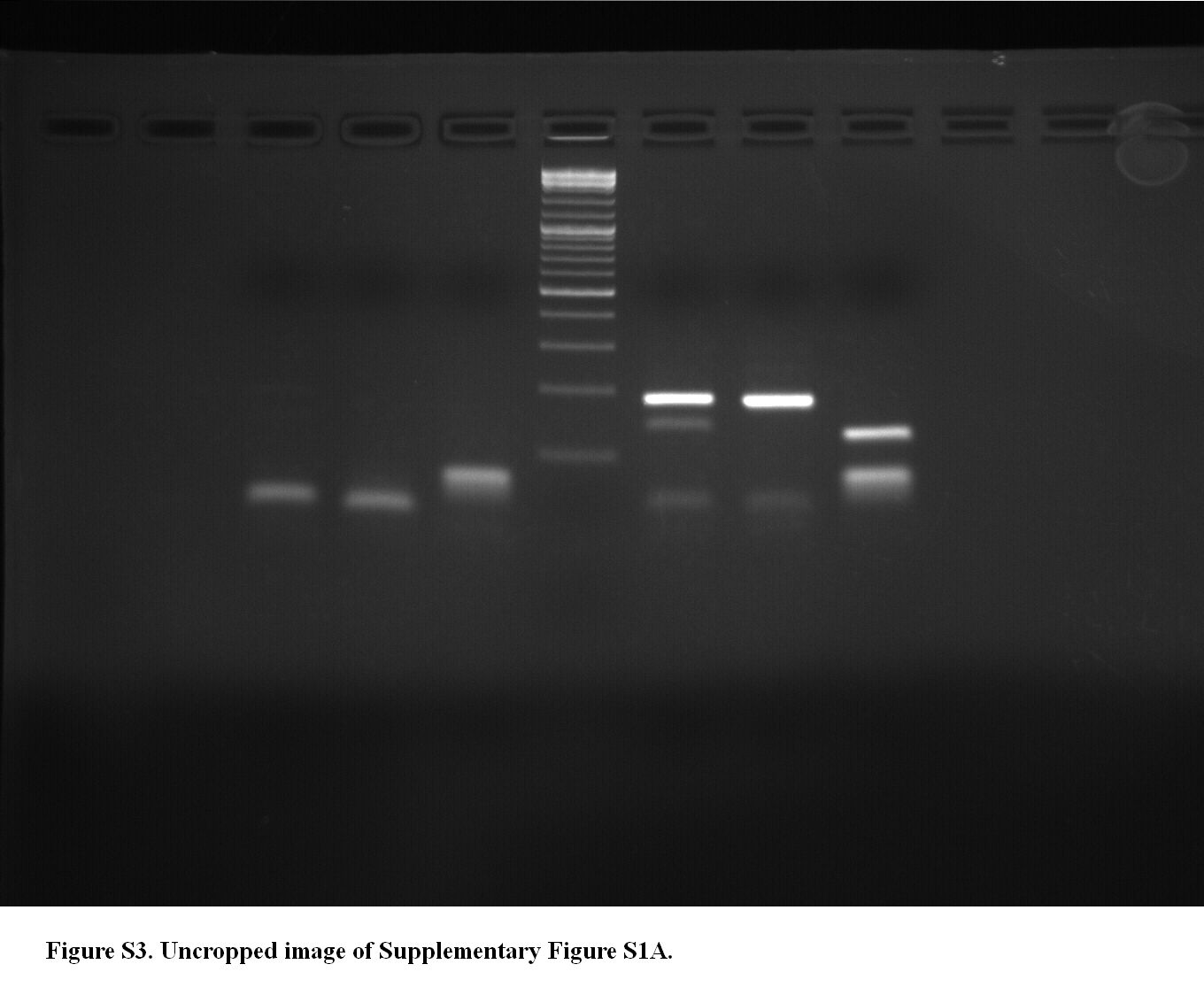

Supplement: Supplementary file 1 — Supplementary Information 1. [file 41598_2022_27287_MOESM1_ESM.jpg]
