# Supplementary material for: Limited water stress modulates expression of circadian clock genes in Brachypodium distachyon roots
Source: Sci Rep. 2023 Jan 23;13:1241. doi: 10.1038/s41598-022-27287-4 (PMC9870971; doi:10.1038/s41598-022-27287-4)
Supplement: Supplementary file 2 — Supplementary Information 2. [file 41598_2022_27287_MOESM2_ESM.pptx]

## Slide 1
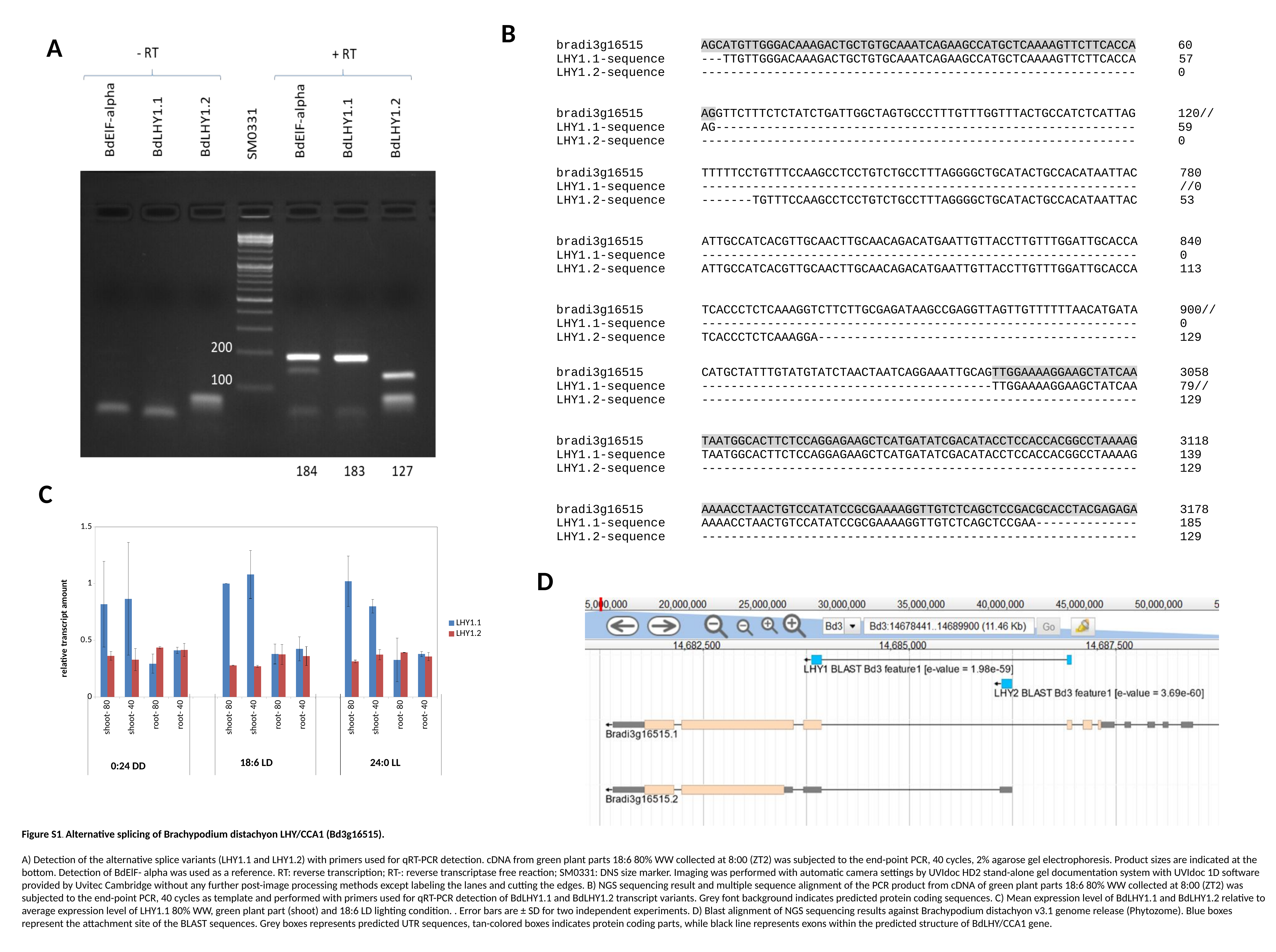

B
A
C
### Chart
| Category | | |
|---|---|---|
| shoot- 80 | 0.8179679961982814 | 0.3621344516218396 |
| shoot- 40 | 0.8657169554898696 | 0.3292250201911404 |
| root- 80 | 0.2932871317103899 | 0.43485074830993203 |
| root- 40 | 0.41145068032929366 | 0.4151163743994499 |
| | None | None |
| shoot- 80 | 1.0 | 0.2775140229401164 |
| shoot- 40 | 1.0796546906028717 | 0.2700789853331594 |
| root- 80 | 0.3786475054793451 | 0.3745147941246915 |
| root- 40 | 0.42454538659873026 | 0.3600665542027672 |
| | None | None |
| shoot- 80 | 1.020086908300229 | 0.31268289889030654 |
| shoot- 40 | 0.7999444487643961 | 0.37282355019341434 |
| root- 80 | 0.32688445013412115 | 0.39152834960051214 |
| root- 40 | 0.37817258940547105 | 0.3559773693204321 |18:6 LD
24:0 LL
0:24 DD
D
Figure S1. Alternative splicing of Brachypodium distachyon LHY/CCA1 (Bd3g16515).
A) Detection of the alternative splice variants (LHY1.1 and LHY1.2) with primers used for qRT-PCR detection. cDNA from green plant parts 18:6 80% WW collected at 8:00 (ZT2) was subjected to the end-point PCR, 40 cycles, 2% agarose gel electrophoresis. Product sizes are indicated at the bottom. Detection of BdElF- alpha was used as a reference. RT: reverse transcription; RT-: reverse transcriptase free reaction; SM0331: DNS size marker. Imaging was performed with automatic camera settings by UVIdoc HD2 stand-alone gel documentation system with UVIdoc 1D software provided by Uvitec Cambridge without any further post-image processing methods except labeling the lanes and cutting the edges. B) NGS sequencing result and multiple sequence alignment of the PCR product from cDNA of green plant parts 18:6 80% WW collected at 8:00 (ZT2) was subjected to the end-point PCR, 40 cycles as template and performed with primers used for qRT-PCR detection of BdLHY1.1 and BdLHY1.2 transcript variants. Grey font background indicates predicted protein coding sequences. C) Mean expression level of BdLHY1.1 and BdLHY1.2 relative to average expression level of LHY1.1 80% WW, green plant part (shoot) and 18:6 LD lighting condition. . Error bars are ± SD for two independent experiments. D) Blast alignment of NGS sequencing results against Brachypodium distachyon v3.1 genome release (Phytozome). Blue boxes represent the attachment site of the BLAST sequences. Grey boxes represents predicted UTR sequences, tan-colored boxes indicates protein coding parts, while black line represents exons within the predicted structure of BdLHY/CCA1 gene.

## Slide 2
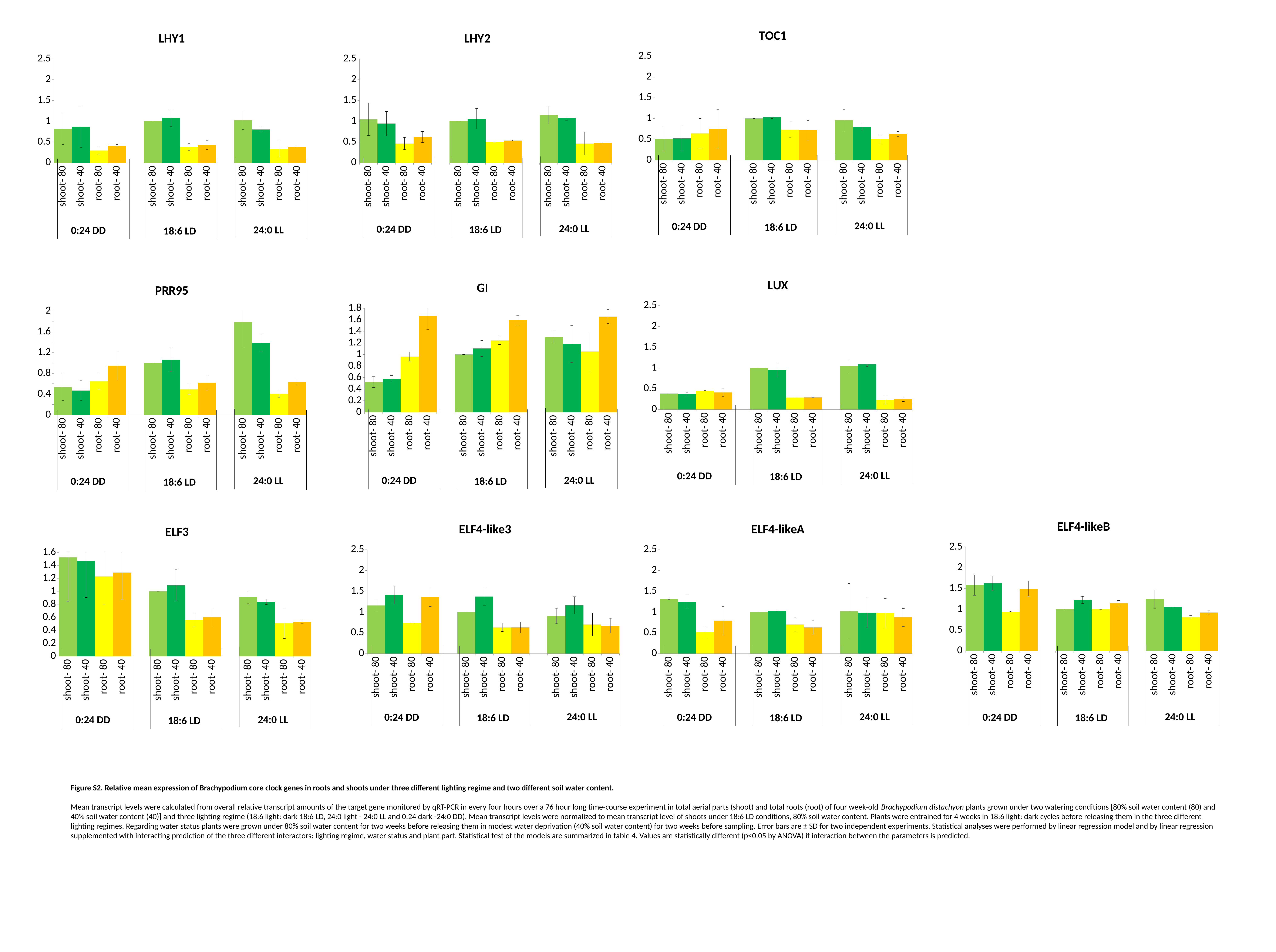

### Chart:
| Category | |
|---|---|
| shoot- 80 | 0.5069579929311502 |
| shoot- 40 | 0.5206112394009197 |
| root- 80 | 0.6424053503211926 |
| root- 40 | 0.7514702710924995 |
| | None |
| shoot- 80 | 1.0 |
| shoot- 40 | 1.029846183807955 |
| root- 80 | 0.7309072655683566 |
| root- 40 | 0.7168690515967269 |
| | None |
| shoot- 80 | 0.9536462240013441 |
| shoot- 40 | 0.7942851009298663 |
| root- 80 | 0.5049469934618611 |
| root- 40 | 0.6245909151103334 |
### Chart:
| Category | |
|---|---|
| shoot- 80 | 0.8179679961982815 |
| shoot- 40 | 0.8657169554898696 |
| root- 80 | 0.29328713171039034 |
| root- 40 | 0.4114506803292931 |
| | None |
| shoot- 80 | 1.0 |
| shoot- 40 | 1.0796546906028717 |
| root- 80 | 0.3786475054793457 |
| root- 40 | 0.4245453865987303 |
| | None |
| shoot- 80 | 1.0200869083002289 |
| shoot- 40 | 0.7999444487643959 |
| root- 80 | 0.32688445013412154 |
| root- 40 | 0.37817258940547194 |
### Chart:
| Category | |
|---|---|
| shoot- 80 | 1.0436147715951263 |
| shoot- 40 | 0.9405641250170509 |
| root- 80 | 0.46164720772169776 |
| root- 40 | 0.6188910057141669 |
| | None |
| shoot- 80 | 1.0 |
| shoot- 40 | 1.0551323464224078 |
| root- 80 | 0.4969736757613761 |
| root- 40 | 0.5350504059601816 |
| | None |
| shoot- 80 | 1.1456376070142298 |
| shoot- 40 | 1.0700584848831776 |
| root- 80 | 0.4620670142553319 |
| root- 40 | 0.48356503853065247 |24:0 LL
0:24 DD
18:6 LD
24:0 LL
0:24 DD
18:6 LD
24:0 LL
0:24 DD
18:6 LD
### Chart:
| Category | |
|---|---|
| shoot- 80 | 0.3855097513187399 |
| shoot- 40 | 0.3683206914649926 |
| root- 80 | 0.4518454613941 |
| root- 40 | 0.4099887537981896 |
| | None |
| shoot- 80 | 1.0 |
| shoot- 40 | 0.9518171410490885 |
| root- 80 | 0.2883481902768463 |
| root- 40 | 0.2917337939778144 |
| | None |
| shoot- 80 | 1.0493230152508646 |
| shoot- 40 | 1.0869414436838167 |
| root- 80 | 0.2282467847209286 |
| root- 40 | 0.2471556192094528 |
### Chart:
| Category | |
|---|---|
| shoot- 80 | 0.5221322858795673 |
| shoot- 40 | 0.5813404832521457 |
| root- 80 | 0.9658721556046529 |
| root- 40 | 1.6731739516058932 |
| | None |
| shoot- 80 | 1.0 |
| shoot- 40 | 1.104560923827385 |
| root- 80 | 1.2432666780594066 |
| root- 40 | 1.5938276609840658 |
| | None |
| shoot- 80 | 1.3037485135278875 |
| shoot- 40 | 1.1826684194518318 |
| root- 80 | 1.0514634330308033 |
| root- 40 | 1.6582402523483957 |
### Chart:
| Category | |
|---|---|
| shoot- 80 | 0.5309549498252504 |
| shoot- 40 | 0.46787049678181375 |
| root- 80 | 0.6500823597797358 |
| root- 40 | 0.9486375190441707 |
| | None |
| shoot- 80 | 1.0 |
| shoot- 40 | 1.0612526623534313 |
| root- 80 | 0.4953252750266179 |
| root- 40 | 0.6223566951748167 |
| | None |
| shoot- 80 | 1.7867447318150704 |
| shoot- 40 | 1.3799673045578809 |
| root- 80 | 0.40807548921137154 |
| root- 40 | 0.6331448576361124 |24:0 LL
0:24 DD
18:6 LD
24:0 LL
0:24 DD
18:6 LD
24:0 LL
0:24 DD
18:6 LD
### Chart:
| Category | |
|---|---|
| shoot- 80 | 1.584725898687147 |
| shoot- 40 | 1.630042591991572 |
| root- 80 | 0.9443422852531183 |
| root- 40 | 1.4983259043902701 |
| | None |
| shoot- 80 | 1.0 |
| shoot- 40 | 1.2234709870617726 |
| root- 80 | 0.9996683581897003 |
| root- 40 | 1.1452991678614954 |
| | None |
| shoot- 80 | 1.2453998222653337 |
| shoot- 40 | 1.056167267779569 |
| root- 80 | 0.8096617642447511 |
| root- 40 | 0.9229598092358386 |
### Chart:
| Category | |
|---|---|
| shoot- 80 | 1.1562815130919464 |
| shoot- 40 | 1.4122748574282578 |
| root- 80 | 0.7436343310147534 |
| root- 40 | 1.359261246818528 |
| | None |
| shoot- 80 | 1.0 |
| shoot- 40 | 1.3703839301176615 |
| root- 80 | 0.6284042329032299 |
| root- 40 | 0.6319165342723145 |
| | None |
| shoot- 80 | 0.9030823444481999 |
| shoot- 40 | 1.162078836618642 |
| root- 80 | 0.7023615465529273 |
| root- 40 | 0.6704855805049224 |
### Chart:
| Category | |
|---|---|
| shoot- 80 | 1.3138568009732794 |
| shoot- 40 | 1.2445560537509262 |
| root- 80 | 0.5169000832701246 |
| root- 40 | 0.7914956650501361 |
| | None |
| shoot- 80 | 1.0 |
| shoot- 40 | 1.025588314393071 |
| root- 80 | 0.6994431301839196 |
| root- 40 | 0.6304836084176003 |
| | None |
| shoot- 80 | 1.018018788643322 |
| shoot- 40 | 0.9817563186033513 |
| root- 80 | 0.9710117585949078 |
| root- 40 | 0.8689898857354437 |
### Chart:
| Category | |
|---|---|
| shoot- 80 | 1.5219940498642655 |
| shoot- 40 | 1.465350456731722 |
| root- 80 | 1.2302634048573695 |
| root- 40 | 1.2906077837160965 |
| | None |
| shoot- 80 | 1.0 |
| shoot- 40 | 1.0926244719712082 |
| root- 80 | 0.5585742966712983 |
| root- 40 | 0.6019272874811896 |
| | None |
| shoot- 80 | 0.9129451599330018 |
| shoot- 40 | 0.8390471757361643 |
| root- 80 | 0.5094642927277016 |
| root- 40 | 0.5295115735546395 |24:0 LL
0:24 DD
18:6 LD
24:0 LL
0:24 DD
18:6 LD
24:0 LL
0:24 DD
18:6 LD
24:0 LL
0:24 DD
18:6 LD
Figure S2. Relative mean expression of Brachypodium core clock genes in roots and shoots under three different lighting regime and two different soil water content.
Mean transcript levels were calculated from overall relative transcript amounts of the target gene monitored by qRT-PCR in every four hours over a 76 hour long time-course experiment in total aerial parts (shoot) and total roots (root) of four week-old Brachypodium distachyon plants grown under two watering conditions [80% soil water content (80) and 40% soil water content (40)] and three lighting regime (18:6 light: dark 18:6 LD, 24:0 light - 24:0 LL and 0:24 dark -24:0 DD). Mean transcript levels were normalized to mean transcript level of shoots under 18:6 LD conditions, 80% soil water content. Plants were entrained for 4 weeks in 18:6 light: dark cycles before releasing them in the three different lighting regimes. Regarding water status plants were grown under 80% soil water content for two weeks before releasing them in modest water deprivation (40% soil water content) for two weeks before sampling. Error bars are ± SD for two independent experiments. Statistical analyses were performed by linear regression model and by linear regression supplemented with interacting prediction of the three different interactors: lighting regime, water status and plant part. Statistical test of the models are summarized in table 4. Values are statistically different (p<0.05 by ANOVA) if interaction between the parameters is predicted.

## Slide 3
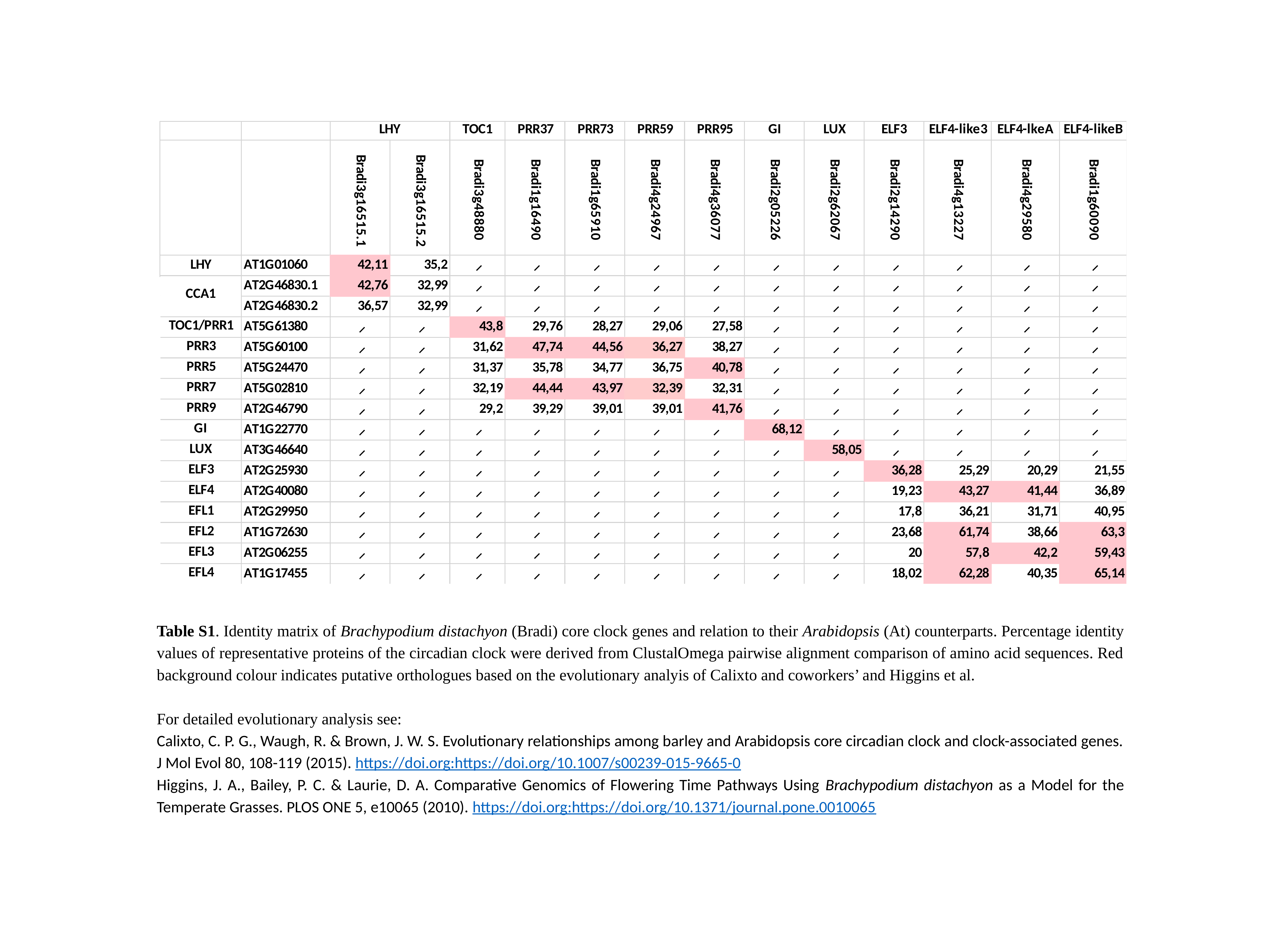

Table S1. Identity matrix of Brachypodium distachyon (Bradi) core clock genes and relation to their Arabidopsis (At) counterparts. Percentage identity values of representative proteins of the circadian clock were derived from ClustalOmega pairwise alignment comparison of amino acid sequences. Red background colour indicates putative orthologues based on the evolutionary analyis of Calixto and coworkers’ and Higgins et al.
For detailed evolutionary analysis see:
Calixto, C. P. G., Waugh, R. & Brown, J. W. S. Evolutionary relationships among barley and Arabidopsis core circadian clock and clock-associated genes. J Mol Evol 80, 108-119 (2015). https://doi.org:https://doi.org/10.1007/s00239-015-9665-0
Higgins, J. A., Bailey, P. C. & Laurie, D. A. Comparative Genomics of Flowering Time Pathways Using Brachypodium distachyon as a Model for the Temperate Grasses. PLOS ONE 5, e10065 (2010). https://doi.org:https://doi.org/10.1371/journal.pone.0010065

## Slide 4
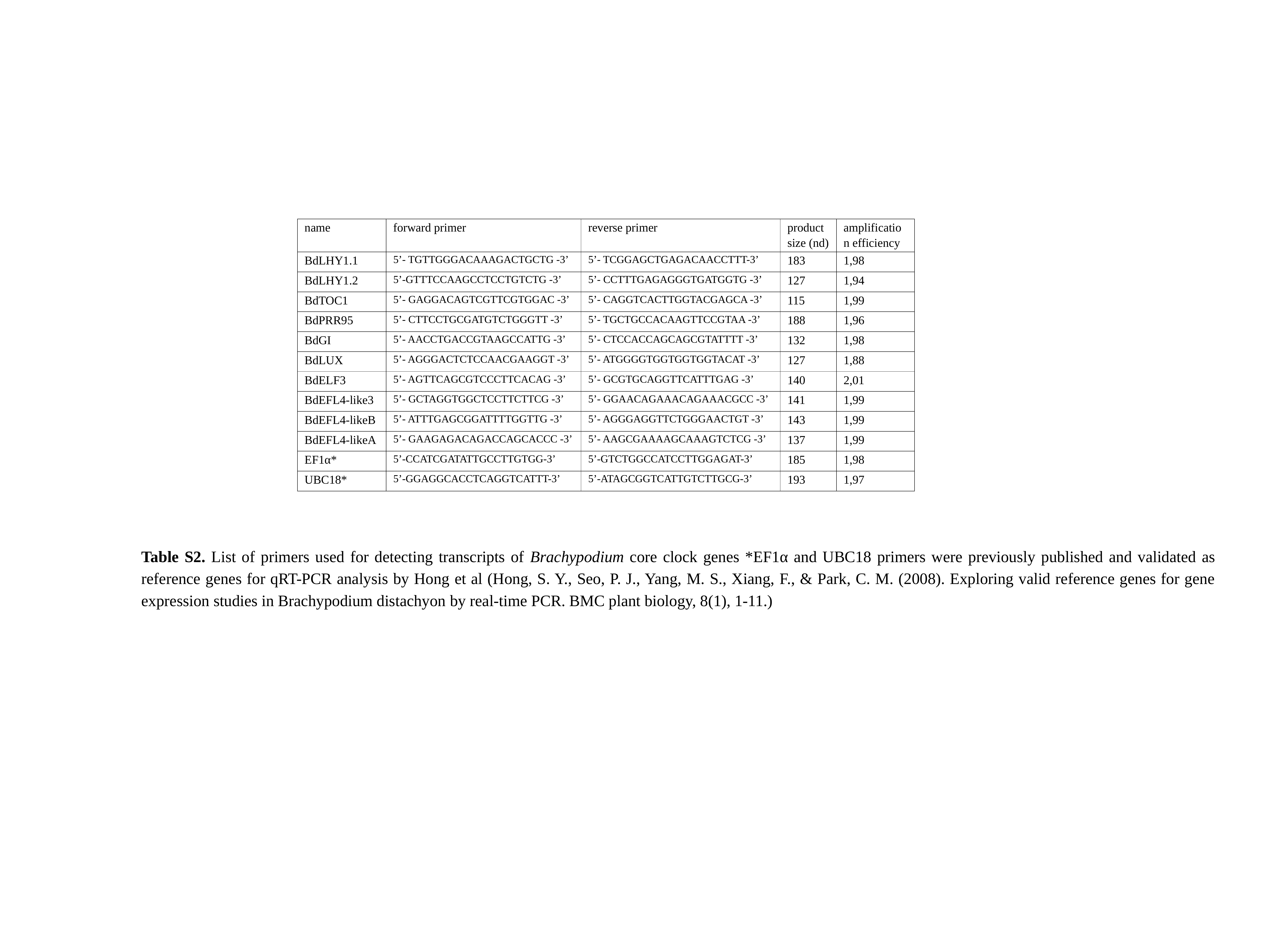

| name | forward primer | reverse primer | product size (nd) | amplification efficiency |
| --- | --- | --- | --- | --- |
| BdLHY1.1 | 5’- TGTTGGGACAAAGACTGCTG -3’ | 5’- TCGGAGCTGAGACAACCTTT-3’ | 183 | 1,98 |
| BdLHY1.2 | 5’-GTTTCCAAGCCTCCTGTCTG -3’ | 5’- CCTTTGAGAGGGTGATGGTG -3’ | 127 | 1,94 |
| BdTOC1 | 5’- GAGGACAGTCGTTCGTGGAC -3’ | 5’- CAGGTCACTTGGTACGAGCA -3’ | 115 | 1,99 |
| BdPRR95 | 5’- CTTCCTGCGATGTCTGGGTT -3’ | 5’- TGCTGCCACAAGTTCCGTAA -3’ | 188 | 1,96 |
| BdGI | 5’- AACCTGACCGTAAGCCATTG -3’ | 5’- CTCCACCAGCAGCGTATTTT -3’ | 132 | 1,98 |
| BdLUX | 5’- AGGGACTCTCCAACGAAGGT -3’ | 5’- ATGGGGTGGTGGTGGTACAT -3’ | 127 | 1,88 |
| BdELF3 | 5’- AGTTCAGCGTCCCTTCACAG -3’ | 5’- GCGTGCAGGTTCATTTGAG -3’ | 140 | 2,01 |
| BdEFL4-like3 | 5’- GCTAGGTGGCTCCTTCTTCG -3’ | 5’- GGAACAGAAACAGAAACGCC -3’ | 141 | 1,99 |
| BdEFL4-likeB | 5’- ATTTGAGCGGATTTTGGTTG -3’ | 5’- AGGGAGGTTCTGGGAACTGT -3’ | 143 | 1,99 |
| BdEFL4-likeA | 5’- GAAGAGACAGACCAGCACCC -3’ | 5’- AAGCGAAAAGCAAAGTCTCG -3’ | 137 | 1,99 |
| EF1α\* | 5’-CCATCGATATTGCCTTGTGG-3’ | 5’-GTCTGGCCATCCTTGGAGAT-3’ | 185 | 1,98 |
| UBC18\* | 5’-GGAGGCACCTCAGGTCATTT-3’ | 5’-ATAGCGGTCATTGTCTTGCG-3’ | 193 | 1,97 |
Table S2. List of primers used for detecting transcripts of Brachypodium core clock genes *EF1α and UBC18 primers were previously published and validated as reference genes for qRT-PCR analysis by Hong et al (Hong, S. Y., Seo, P. J., Yang, M. S., Xiang, F., & Park, C. M. (2008). Exploring valid reference genes for gene expression studies in Brachypodium distachyon by real-time PCR. BMC plant biology, 8(1), 1-11.)

## Slide 5
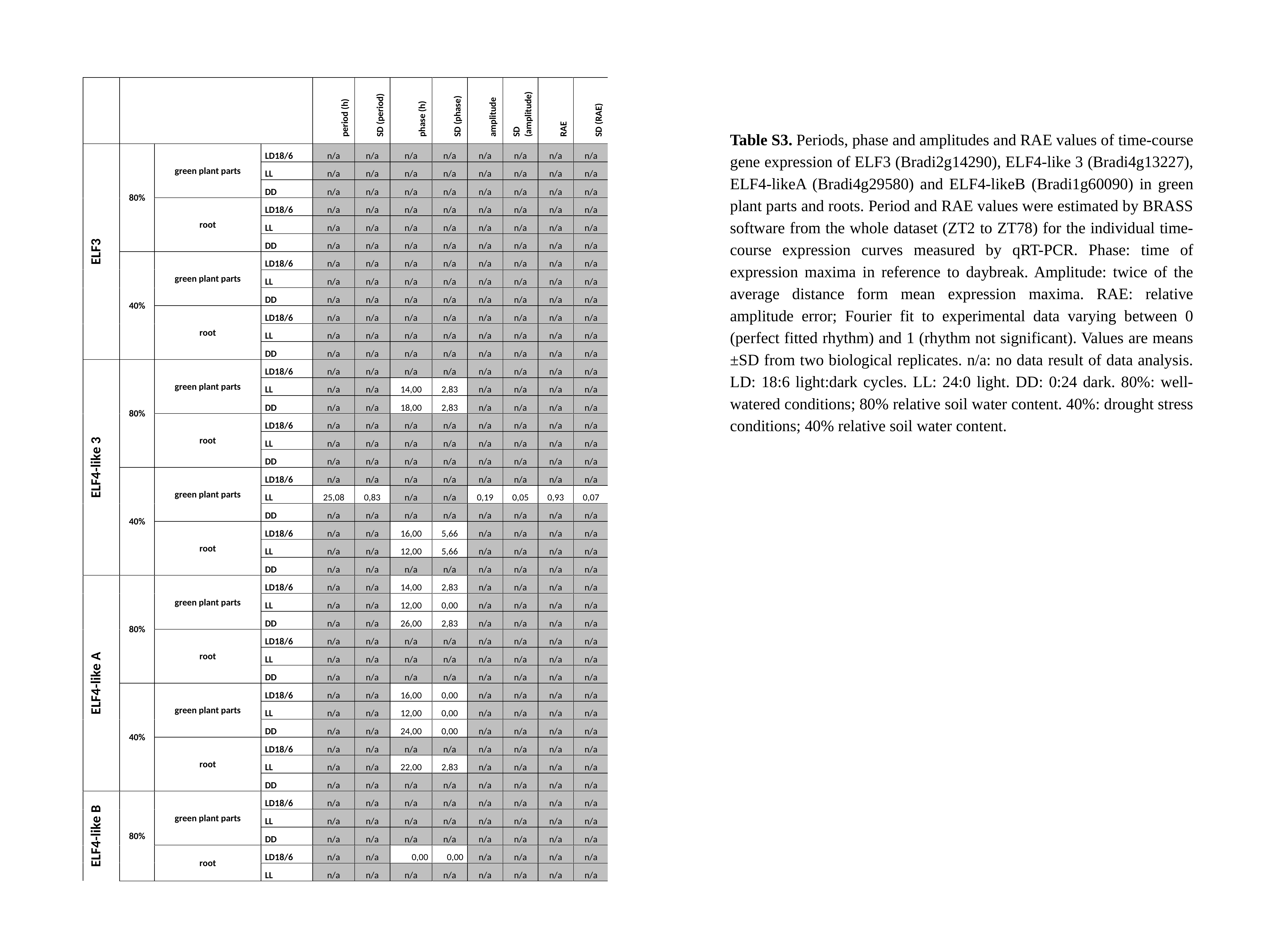

Table S3. Periods, phase and amplitudes and RAE values of time-course gene expression of ELF3 (Bradi2g14290), ELF4-like 3 (Bradi4g13227), ELF4-likeA (Bradi4g29580) and ELF4-likeB (Bradi1g60090) in green plant parts and roots. Period and RAE values were estimated by BRASS software from the whole dataset (ZT2 to ZT78) for the individual time-course expression curves measured by qRT-PCR. Phase: time of expression maxima in reference to daybreak. Amplitude: twice of the average distance form mean expression maxima. RAE: relative amplitude error; Fourier fit to experimental data varying between 0 (perfect fitted rhythm) and 1 (rhythm not significant). Values are means ±SD from two biological replicates. n/a: no data result of data analysis. LD: 18:6 light:dark cycles. LL: 24:0 light. DD: 0:24 dark. 80%: well-watered conditions; 80% relative soil water content. 40%: drought stress conditions; 40% relative soil water content.
